# Combined and Individual Effects of *Chlorella* sp. and Bacterial Probiotics in Biofloc Systems for White Shrimp (*Penaeus vannamei*)

**DOI:** 10.3390/ani16162469

**Published:** 2026-08-08

**Authors:** Tatiana Cascales-Martos, Jessica Brol, Silvia Martínez-Llorens, Ana Tomás-Vidal, M. Jover-Cerdá, F. J. Moyano, D. S. Peñaranda

**Affiliations:** 1 Aquaculture and Biodiversity Research Group, Universitat Politècnica de València, Camí de Vera s/n, 46022 València, Spain; tcasmar@upvnet.upv.es (T.C.-M.); jbrol@doctor.upv.es (J.B.); silmarll@dca.upv.es (S.M.-L.); atomasv@dca.upv.es (A.T.-V.); mjover@dca.upv.es (M.J.-C.); 2 Mediterranean Agrosystems and Agri-Food Biotechnology Research Centre (CIAMBITAL), Department of Biology and Geology, University of Almería, 04120 Almería, Spain; fjmoyano@ual.es

**Keywords:** biofloc, probiotic, *P. vannamei*, *Chlorella* sp., *Bacillus*, intestinal microbiota, bioremediation

## Abstract

This study aimed to evaluate the effects of establishing a population of the green microalgae *Chlorella* sp. on different key aspects of a biofloc culture of *Penaeus vannamei* in comparison with those obtained with a commercial probiotic. The aspects evaluated were shrimp zootechnical performance, nutritional composition of biofloc and microbial populations of shrimp intestinal microbiota and biofloc. Four treatments were tested: two of them included *Chlorella* sp. alone (M) or in combination to a bacterial probiotic (MP), while the other two included only the probiotic (P) or none of them (C). The presence of *Chlorella* sp. enhanced protein and phosphorus bioavailability in the biofloc and contributed to nutrient bioremediation within the system. However, shrimp growth performance and survival were significantly reduced, likely due to greater and more prolonged nitrate exposure following microalgae inoculation. Although overall nitrate and phosphate concentrations were higher in microalgae treatment, their accumulation over the experimental period was lower in the presence of *Chlorella* sp. In addition, microalgal supplementation reduced the abundance of bacterial groups associated with oligotrophic lifestyles, complex organic matter degradation, and nitrogen cycling in the intestinal microbiota. In contrast, probiotic supplementation increased protein content in shrimp and phosphorus bioavailability in the biofloc, while zootechnical performance remained comparable to the control and was higher than in microalgae treatments. Furthermore, probiotic addition reduced the abundance of Flavobacteriaceae, a bacterial family proposed as a bioindicator of White Feces Syndrome (WFS) in *P. vannamei*.

## Simple Summary

Biofloc technology is an increasingly used strategy for more sustainable shrimp farming. However, new approaches are still needed to improve water quality and animal health. In this study, we evaluated the effects of the green microalga *Chlorella* sp. and a commercial probiotic, applied separately or in combination, in a biofloc system for Pacific white shrimp culture. A single inoculation of *Chlorella* sp. maintained a stable population for approximately 40 days. The microalga contributed to nutrient bioremediation in water and increased the nutritional value of the biofloc, a natural food source for shrimp. However, shrimp reared in microalgae treatments showed lower growth performance and survival, which may have been associated with prolonged exposure to the elevated nitrate levels associated with microalgae inoculation. In contrast, the probiotic and control treatments showed comparable zootechnical performance, and both outperformed the microalgae treatments. Probiotic supplementation also reduced the abundance of Flavobacteriaceae, a bacterial family proposed as a bioindicator of White Feces Syndrome (WFS). Overall, these findings suggest that microalgae have considerable potential to improve the sustainability of biofloc systems, provided that the inoculation protocol should be optimized, and confirm the probiotic as an effective strategy to enhance shrimp productivity and health.

## 1. Introduction

Biofloc technology (BFT) is considered a sustainable system for shrimp culture due to its zero or minimal water exchange, which reduces the environmental impact of production while providing additional biosecurity [1,2]. In addition, BFT can reduce the dependence on artificial feed through the continuous availability of microbial biomass as a supplementary food source, while also improving animal health and immune status [3].

Nevertheless, the super-intensive stocking conditions commonly applied in biofloc systems may induce chronic stress, which can negatively affect shrimp welfare, growth performance, feed efficiency, and survival. High stocking densities increase competition for space and dissolved oxygen, elevate organic matter accumulation, and may lead to the deterioration of water quality parameters such as ammonia, nitrite, and suspended solids. These conditions can trigger physiological stress responses, suppress immune function, and increase susceptibility to disease outbreaks, ultimately compromising production performance [4,5,6]. To mitigate these drawbacks, several management and nutritional strategies have been evaluated, including optimization of stocking density, improvement of aeration and solids control, manipulation of the carbon-to-nitrogen (C:N) ratio, and the use of functional additives such as probiotics, prebiotics, phytogenics, and immunostimulants [7].

The use of beneficial microorganisms, particularly probiotic bacteria and microalgae, has emerged as a sustainable strategy to improve water quality, enhance shrimp health, and reduce disease outbreaks in aquaculture systems [8,9]. In biofloc systems, several naturally occurring probiotic genera, including *Bacillus*, *Lactobacillus*, *Pseudomonas*, and *Shewanella*, contribute to nutrient recycling, pathogen inhibition, and immune stimulation [10]. Among them, *Bacillus* spp. are currently one of the most widely used probiotics in commercial aquaculture due to their ability to produce digestive enzymes, improve nutrient utilization, stimulate immune responses, and alleviate culture-related stress in *P. vannamei* [11,12,13,14]. In particular, *Bacillus subtilis* and *Bacillus licheniformis* have been shown to promote bioremediation processes and suppress opportunistic pathogens such as *Vibrio* spp., while probiotic supplementation in biofloc systems further enhances the proliferation of beneficial microbial populations, contributing to improved shrimp health and growth performance [9,11].

Additionally, it has been demonstrated that probiotics are able to establish and proliferate within the gastrointestinal tract, thereby contributing to the effective modulation of multiple biological processes in the aquatic host [9]. Previous studies reported that *Lactobacillus plantarum* isolated from the intestinal tract of *P. vannamei* reduced *Vibrio* counts in both the culture water and shrimp digestive tract when applied in a biofloc system [15]. In addition, potential probiotic isolates obtained from biofloc communities have been evaluated as dietary supplements, improving growth performance and microbial balance in shrimp farming systems [16].

Microalgae have also shown positive effects on health status, growth performance, and water quality [17]. However, the incorporation of microalgae into biofloc systems has traditionally been limited due to low light penetration caused by the high concentration of suspended solids. Despite this limitation, their application in biofloc environments has increased in recent years because of their beneficial effects on water quality, antioxidant capacity, immune response, and pathogen control [18,19]. Species such as *Grammatophora* sp., *Navicula* sp., *Schizochytrium* sp., and *Tetraselmis suecica* have been associated with a reduction in *Vibrio* spp. levels in biofloc [20,21]. In addition, algal-derived long-chain polyunsaturated fatty acids, such as eicosapentaenoic acid (EPA), together with algal sterols and carotenoids (lutein, zeaxanthin, and astaxanthin), exhibit antibacterial properties and contribute to increased survival of aquatic organisms during pathogenic challenges, highlighting their potential as functional ingredients in aquaculture nutrition [21].

*Chlorella* sp. is characterized by its elevated protein concentration and lipid accumulation capacity [22,23,24]. When incorporated into biofloc systems, *Chlorella* sp. contributes to improved floc composition, enriching it with the content of arachidonic acid (ARA) and EPA [25,26]. It has also been reported to exert positive effects on the overall biofloc community, increasing also the biodiversity of the bacterial community [27]. Other previous studies have also demonstrated beneficial effects of *Chlorella* supplementation on the growth performance, immune response, antioxidant capacity, and disease resistance of *P. vannamei*. For instance, dietary or water supplementation with *Chlorella* spp. has been associated with enhanced weight gain, improved feed conversion ratio, increased activities of immune-related enzymes, and greater resistance against *Vibrio* infections [17,28,29]. However, in biofloc systems, the accumulation of nitrogen and phosphorus may promote the proliferation of undesirable phytoplankton, including filamentous cyanobacteria, particularly under outdoor conditions [30]. In this context, the incorporation of microalgae, such as *Chlorella* sp., may contribute to the removal of dissolved nutrients through their uptake, thereby limiting cyanobacterial dominance via resource competition, improving water quality, and supporting water reuse or reducing the environmental impact of effluent discharge [25].

Therefore, the present study aimed to evaluate the individual and combined effects of *Chlorella* sp. and commercial bacterial probiotics (*Bacillus subtilis* and *Bacillus licheniformis*) in a super-intensive biofloc system for *Penaeus vannamei*. Special emphasis was put on the effects on biofloc nutritional quality and microbiota composition, as well as on shrimp intestinal microbiota, nutritional composition, and zootechnical performance.

## 2. Materials and Methods

### 2.1. Ethics Approval

Crustaceans are not included within the scope of Council Directive 2010/63/EU; therefore, ethical approval from an institutional animal care and use committee was not required. Nevertheless, all experimental procedures involving animals were conducted in accordance with applicable national regulations and were classified as mild according to Annex IX of Royal Decree 53/2013. Animals were euthanized by rapid chilling through immersion in ice water to obtain tissue samples [31]. The experiments were carried out at the Aquaculture Laboratory of the Universitat Politècnica de València, an accredited facility (authorization number ES 46 250 0001091).

### 2.2. Animals and Microalgae Culture

*Chlorella* sp. was provided by the Species Conservation Laboratory of the University of Murcia Aquarium (Murcia, Spain), where it was maintained as an auxiliary culture. Initially, the culture was maintained at 25 °C and a salinity of 29 PSU. Prior to the experiment, the microalgae were acclimated in a controlled culture chamber, where salinity, temperature, and light intensity were progressively adjusted to the experimental conditions. Culture conditions were maintained at 28 °C, a salinity of 21 PSU, continuous illumination (3500–4000 lux), and constant aeration. Microalgae were cultured in batch systems through a stepwise scale-up process. Initially, cultures were grown in 1 L flasks for 5–7 days. Upon reaching the exponential growth phase, they were transferred to 6 L round flasks and subsequently scaled up to 50 L plastic bag photobioreactors. Continuous aeration was supplied using sterile air via air stones, and culture media were prepared with previously autoclaved water. Microalgae were fertilized with a commercial nutrient solution for marine microalgae (Easyphyt^®^, Easy Reef, El Puerto de Santa María, Cádiz, Spain) at a concentration of 3 mL L^−1^. The *Chlorella* sp. culture was non-axenic and therefore contained its naturally associated bacterial community.

Shrimp larvae (*Penaeus vannamei*; 0.001 ± 0.002 g) were obtained from White Panther Company (Austria). Upon arrival, the larvae were at a salinity of 33 PSU and were gradually acclimated to 21 PSU over a two-week period. During acclimation, shrimp were continuously fed a commercial diet (Le Gouessant, Lamballe-Armor, France) using automatic feeders. Water quality parameters, including salinity, dissolved oxygen, pH, and temperature, were monitored daily, whereas total ammonia nitrogen (TAN) and nitrite were measured twice per week, and nitrate and phosphate were determined once per week. Juveniles with an average weight of 2.5 ± 0.1 g were subsequently used for the experimental trial.

### 2.3. Experimental Design

**Figure 1:**
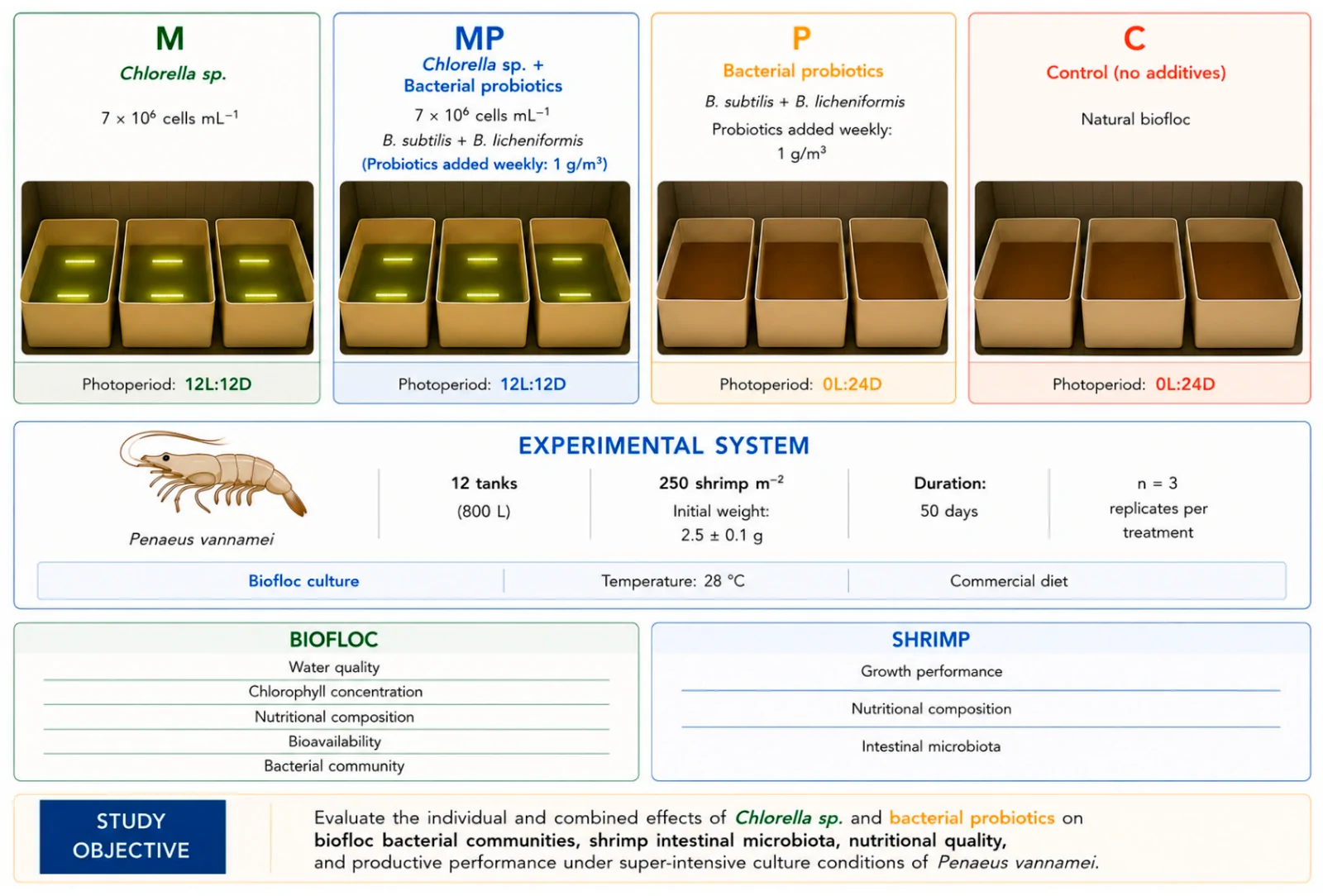
Schematic representation of the experimental design and evaluated variables in the four biofloc treatments: microalgae (*Chlorella* sp.) (M), microalgae (*Chlorella* sp.) + bacterial probiotics (MP), bacterial probiotics (P), and control (C).

Four experimental treatments were designed to evaluate the impact of microalgae and/or probiotic inoculation on bacterial communities present in biofloc and the shrimp intestinal tract (Figure 1):

- A control without probiotics or microalgae (C).
- A treatment on which probiotics were applied at an initial dose of 3 g m^−3^ followed by a weekly maintenance dose of 1 g m^−3^, according to the manufacturer’s recommendations and previous applications in biofloc systems [32] (P). Probiotic bacteria were sourced from the commercial product Sanolife PRO-W^®^ (INVE Aquaculture, Dendermonde, Belgium), being composed of *Bacillus subtilis* and *Bacillus licheniformis* at a concentration of 5 × 10^10^ CFU g^−1^. For probiotic activation, the required amount was suspended in water in a beaker, continuously aerated for 1 h, and subsequently added to the corresponding tanks.
- A microalgae treatment with a single initial inoculation (7 × 10^6^ cells mL^−1^ ) of *Chlorella* sp (M).
- A treatment that combined microalgae and probiotics under the above-described conditions (MP).

A single inoculation of *Chlorella* sp. was intentionally applied at the beginning of the experiment to evaluate its persistence within the biofloc system and to assess whether repeated inoculations were necessary to maintain its biological effects. This approach was selected to simulate a more practical and economically feasible strategy for commercial biofloc production.

Treatments not including microalgae were maintained in darkness (0 L:24 D) to reproduce the conditions commonly used in indoor commercial biofloc systems and minimize possible phytoplankton proliferation. In contrast, the microalgae treatments were maintained under a 12 L:12 D photoperiod, which had previously been identified as the optimal light regime for *Chlorella* sp. proliferation [25]. Illumination was provided by Sera LED X-Change Tube daylight sunrise lamps (16 W) (Sera GmbH, Heinsberg, Germany), emitting natural daylight with a color temperature of 6000–8000 Kelvin. The lamps were placed longitudinally along the tank wall and photoperiod was adjusted for each treatment using automatic timers. Water temperature was maintained at 28 °C using two 200 W electronic glass aquarium heaters with integrated thermostats (20–33 °C adjustment range) in each experimental tank. Continuous aeration was supplied by a 3 kW air blower pump connected to micro-porous air hoses (Aero-tube^®^). The experiment was carried out at a high stocking density (250 animals m^−2^), with 285 animals per experimental unit, each consisting of an 800 L tank with a bottom area of approximately 1.14 m^2^. Shrimp had an initial weight of 2.5 ± 0.1 g and were randomly distributed among 12 tanks (*n* = 3 replicates per experimental group). The experiment lasted 50 days. Samplings aimed to assess variations in body weight and were carried out weekly to adjust the feeding rate based on standard recommendations [33].

### 2.4. Water Quality Analysis

No water renewal was conducted throughout the experiment, and only freshwater was added to compensate for evaporative losses. Daily monitoring of key water quality parameters, including temperature (°C), dissolved oxygen (DO) (mg L^−1^), pH (U.A.) and salinity (PSU), was performed using a multiparametric device (HANNA, HI19829 Model, Woonsocket, RI, USA). Total suspended solids (TSS) were measured weekly as described in [34], which involved filtering water samples through glass-fiber Whatman filters (0.45 μm). Sediment concentrations exceeding 500 mg L^−1^ were reduced by decantation. Alkalinity (mg L^−1^) and phosphate levels (PO_4_^3−^, mg L^−1^) were assessed using spectrophotometric methods (HANNA, HI83203 Model, Woonsocket, RI, USA). Nitrogenous compounds: Ammonium (NH_4_^+^, mg L^−1^) was analyzed based on the protocol described in [35]; nitrite (NO_2_^−^ mg L^−1^) and nitrate (NO_3_^−^ mg L^−1^) determination were carried out based on the use of vanadium (III) chloride as a reducing agent, as first described in [36] and developed by for micro-plate readers using the DLAB SP-UV1100 spectrophotometer (DLAB Scientific Co., Ltd., Beijing, China). Sodium bicarbonate (NaHCO_3_) was used to correct alkalinity levels when they dropped below 150 mg L^−1^. Molasses was used as an exogenous carbon source. The initial dose was calculated according to [37] to achieve a target C:N ratio of 15:1. Thereafter, molasses was applied only when increases (>1 mg L^−1^) in total ammonia nitrogen (TAN) were detected [37].

### 2.5. Biofloc Characterization

#### 2.5.1. Chlorophyll Concentration

The dynamics of the autotrophic community was monitored once weekly by quantifying total chlorophyll concentration. Chlorophylls were measured according to the method of Hansmann [38]. Briefly, a known volume of water sample was filtered through 0.45 µm cellulose acetate filters, which were subsequently stored frozen (−20 °C) for up to one week prior to analysis. Pigment extraction was carried out by immersing the filters in 90% acetone and incubating them in the dark at 4 °C for 24 h. Following extraction, samples were centrifuged at 6500 rpm for 10 min, and the supernatant was collected for spectrophotometric analysis (DLAB SP-UV1100, DLAB Scientific Co., Ltd., Beijing, China). Chlorophyll concentrations (mg L^−1^) were calculated from absorbance readings at 664, 647, and 630 nm using standard equations.

#### 2.5.2. Nutritional Composition

Three biofloc samples per treatment were collected at the end of the experiment by decantation and filtered through a phytoplankton mesh. Samples were pre-dried in an oven at 60–65 °C to facilitate grinding and storage before analysis. At the end of the experiment, six whole shrimp per tank were randomly collected, freeze-dried, ground to a fine homogeneous powder, and stored until analysis. Subsamples were then used to determine dry matter by oven drying at 105 °C to constant weight, ash by incineration at 550 °C for 5 h, crude protein by direct combustion using a LECO CN628 analyzer (LECO Corporation, Geleen, The Netherlands), and total crude lipids after methyl-ether extraction using an ANKOM XT10 Extractor (Macedon, NY, USA). The analyses followed AOAC official methods 934.01, 942.05, 990.03 and 920.39, respectively, and were performed in triplicate. In addition, total phosphorus was determined in biofloc samples by acid digestion followed by spectrophotometric quantification adapted from [39]. Samples included were subjected to wet digestion using concentrated nitric acid and a digestion solution to mineralize organic matter and release inorganic phosphorus. Subsequently, a stop reagent containing molybdate and vanadate was added, forming a yellow-colored complex with orthophosphate. Absorbance was measured at 415 nm, and phosphorus concentration was calculated using a calibration curve prepared from a phosphate (PO_4_^3−^) standard solution.

### 2.6. Shrimp Feeding, Zootechnical Parameters and Analysis

The daily feed rate (DFR) was calculated as follows: Daily feed rate (g) = Biomass (g) × feeding rate (FR, %). Both biomass and FR were adjusted weekly throughout the experiment. To assess biomass while minimizing stress, a random partial sampling of 10% of the shrimp population was conducted each week. The FR was established using a custom feeding table based on [40]. Shrimp were fed using a commercial feed (Le Gouessant Lamballe-Armor, France; Table S1). The daily ration was divided into three equal meals, which were offered at 09:00, 13.00 and 17:00 h.

The animals were weighed at the end of the experiment to calculate zootechnical parameters:

Specific Growth Rate (SGR): 100 × ln (final weight/initial weight)/days [41].
Feed Conversion Ratio (FCR): Feed intake (dry basis) (g)/WG(g) [41].
Survival (S): (final number animals/initial number animals) × 100 (%) [42].
Protein Efficiency Ratio (PER) = Gain weight (g)/consumed protein (g) [43].

For shrimp biochemical analyses, five individuals were randomly collected from each tank at the end of the experimental period, being pooled, freeze-dried, homogenized, and used as a single composite sample for nutritional analyses. Therefore, each tank represented one experimental unit (*n* = 3 per treatment). The parameters and analytical procedures were the same as described for biofloc samples in Section 2.5.2.

### 2.7. In Vitro Digestibility Assay

The differences in the potential bioavailability of the protein fraction in artificial feed and biofloc for shrimp were tested using an in vitro digestion assay specifically developed for this study.

#### 2.7.1. Biological Material

For the analysis, three biofloc samples per treatment were collected at the end of the experiment by decantation and filtered with a phytoplankton mesh. These samples were dried in an oven at 90 °C until complete water removal.

The digestive enzyme extracts used in the assays were obtained from the hepatopancreas of 24 individuals of *P. vannamei* of 8.4 g mean weight collected from culture tanks at the UPV (Valencia, Spain). Non-experimental shrimp were used as enzyme donors to avoid potential variations in enzyme secretion associated with the dietary treatments. In addition, larger individuals were selected to obtain sufficient enzyme extract for all assays and replicates. Preliminary assays performed during optimization of the technique showed no differences between smaller (approximately 2 g) and larger (approximately 10 g) shrimp as enzyme sources.

Shrimp were dissected to obtain the organ that was mechanically homogenized in distilled water (1:3 *w*/*v*.) and then centrifuged (12,000× *g*; 15 min; 4 °C) to obtain a clear supernatant that was distributed in aliquots and stored at −20 °C until used in the assay. Trypsin activity was determined in the extract using Nα-Benzoyl-DL-arginine p-nitroanilide (BAPNA) 50 mM.

#### 2.7.2. Assay Conditions

Assay conditions considered several key parameters: pH, optimal concentrations of Na^+^ and Ca^2+^ ions, physiological enzyme:substrate ratios and duration of the assay. The operative pH (8.0) was based on previous determinations of optimal pH for trypsin of *P. vannamei*. The physiologically based enzyme:substrate ratio used was 0.038 mU trypsin mg^−1^, and it was established considering, on one hand, the average total trypsin activity measured in the sampled individuals and, on the other, the estimated amount of protein ingested per day of shrimp of such size when consuming 8% l.w. of a feed with 35% CP. The in vitro assay was used for the comparative assessment of bioavailability among treatments; therefore, small variations in the enzyme ratio do not affect the relative comparisons among treatments. The reaction time was fixed at 5 h and the temperature was maintained at 28 °C.

#### 2.7.3. Bioreactors

Semi-permeable membrane bioreactors were employed in the assays. Each bioreactor consisted of two chambers separated by a 3.5 kDa MWCO membrane (ZelluTrans, Carl Roth GmbH, Karlsruhe, Germany). The digestion was initiated by adjusting the pH to the target value with 0.1 M borate buffer (pH 8.0, containing 60 mM Na^+^ and 24 mM Ca^2+)^ and subsequently adding the hepatopancreas enzyme extract. Small peptides released and permeating across the membrane into the lower chamber were collected at designated time intervals (1, 2, 3.5 and 5 h) via a constant flow of the same alkaline buffer. The total amount of amino acids present in the samples was quantified using orthophthaldehyde [44]. The entire experimental setup, comprising multiple bioreactor units, was maintained within a thermal chamber at a temperature of 28 °C. All assays were performed in triplicate. Additionally, a blank, utilizing heat-inactivated enzyme extracts, was included for each ingredient to quantify basal free amino acid release not attributable to enzymatic hydrolysis. Results are presented as cumulative product release over time, as well as the total mass of released amino acids expressed as a percentage of the total protein.

### 2.8. Biofloc and Shrimp Intestine Microbiome Characterization

#### 2.8.1. DNA Extraction, 16S rRNA Gene Amplification and PacBio Sequencing

For microbiome analysis, one homogeneous biofloc sample per tank was collected at the beginning and at the end of the experiment. For intestinal microbiota analyses, two shrimp were sampled from each experimental tank (six shrimp per treatment). All samples were preserved in nitrogen liquid and subsequently stored at −80 °C until DNA extraction.

Biofloc DNA was extracted using the NucleoSpin^®^ eDNA Water kit (MACHEREY-NAGEL, Düren, Germany, Ref.: 740402.50); meanwhile, DNA from intestine samples were isolated using the QIAamp PowerFecal Pro DNA Kit (Qiagen, Hilden, Germany, Ref.: 51804) following both the manufacturer’s instructions. Samples were lysed in PowerBead Tubes using a TissueLyser II (Qiagen, Hilden, Germany) for 2 min at 25 Hz. The DNA quality was determined using Nanodrop ND-1000 spectrophotometer (Thermo Scientific, Wilmington, DE, USA) and DNA was quantified using Qubit fluorometer (Life Technologies, Paisley, UK). DNA was stored at −20 °C until the PCR amplification.

Libraries of 16S ribosomal RNA gene amplicons were prepared using universal primers 27F (AGRGTTYGATYMTGGCTCAG) and 1492R (RGYTACCTTGTTACGACTT), both tagged with PacBio-specific barcodes to enable multiplex sequencing and minimize chimera formation. PCR amplification was performed with the KAPA HiFi Hot Start DNA Polymerase kit (KAPA Biosystems, Wilmington, MA, USA, Ref.: 7958935001) under the following conditions: denaturation at 98 °C for 20 s, annealing at 57 °C for 30 s, and extension at 72 °C for 75 s for a total of 20 cycles. Post-amplification quality control was performed using a Fragment Analyzer (Agilent Technologies, Santa Clara, CA, USA), and amplicons were pooled in equimolar concentrations for library preparation with the Kinnex 16S rRNA kit (PacBio, Menlo Park, CA, USA, Ref.: 103-072-100), following the Preparing Kinnex Libraries from 16S rRNA Amplicons protocol (PacBio, Part Number 103-238-800 REV02).

The library pool concentration and average size were assessed using the Qubit HS DNA kit (Invitrogen, Carlsbad, CA, USA) and the Fragment Analyzer (Agilent Technologies, Santa Clara, CA, USA), respectively. Final preparation included primer annealing and polymerase binding steps using the Sequel II Binding Kit 3.2 (PacBio, Menlo Park, CA, USA, Ref.: 102-333-300) and the Sequel II DNA Internal Control Complex 1.0 (PacBio, Menlo Park, CA, USA, Ref.: 101-717-600). Libraries were sequenced on the Sequel II system (PacBio) using the Sequel II Sequencing Kit 2.0 (PacBio, Menlo Park, CA, USA, Ref.: 101-820-200).

#### 2.8.2. Bioinformatic Analysis

Bioinformatic analysis of the PacBio 16S rRNA gene sequencing data (https://www.lifescipy.net/PacBio/, accessed on 17 May 2026) was performed using QIIME 2 (version 2021.4). Raw circular consensus sequences were processed with the DADA2 plugin for quality filtering, denoising, and chimera removal. High-quality sequences were then resolved into amplicon sequence variants (ASVs). Taxonomic assignment was performed using the classify-sklearn naïve Bayes classifier implemented in the feature-classifier plugin against the SILVA v138 database. Sequences not assigned to any taxon or classified as Eukaryota, Archaea, or only at the domain level (Bacteria) were removed. The resulting feature table was annotated with taxonomic information and exported for subsequent statistical analyses.

### 2.9. Statistical Analysis

Statistical analyses were performed using SPSS Statistics 28 (IBM Corp., Armonk, NY, USA) and RStudio (v4.3.2). Zootechnical performance, water quality parameters, biofloc composition, nutrient bioavailability, and shrimp composition data were analyzed using one-way ANOVA after verifying normality (Shapiro–Wilk test) and homogeneity of variances (Levene’s test). When significant differences were detected (*p* < 0.05), means were compared using the Student–Newman–Keuls post hoc test. Chlorophyll concentration data were analyzed using a two-factor ANOVA considering treatment and sampling time as fixed factors.

For microbiota analyses, alpha diversity indices (Shannon, Simpson, and Chao1) were calculated for each sample and compared among treatments using the non-parametric Kruskal–Wallis test. When significant differences were detected (*p* < 0.05), pairwise comparisons were performed using Dunn’s post hoc test with Benjamini–Hochberg correction for multiple testing. Beta diversity was assessed using Bray–Curtis dissimilarities and visualized through principal coordinates analysis (PCoA). Prior to beta diversity analyses, taxa with mean relative abundances below 1% were removed. For intestinal microbiota, two shrimp were randomly collected from each replicate tank and considered subsamples. To avoid pseudoreplication, the experimental tank was considered the experimental unit (*n* = 3 per treatment), and the taxonomic relative abundances of the two shrimp from each tank were averaged prior to calculating Bray–Curtis dissimilarities and performing the beta diversity analyses. Differences in community composition among treatments were evaluated using PERMANOVA (adonis2 function, vegan package) based on Bray–Curtis distances with 999 permutations. Differential abundance analyses at the phylum and family levels were performed using the ALDEx2 package (v1.30.0). Taxa with a mean relative abundance below 1% or lacking taxonomic assignment were removed prior to analysis. Abundance matrices were matched with metadata and transformed using centered log-ratio (CLR) transformation with 128 Monte Carlo instances (mc.samples = 128, denom = “all”). Pairwise comparisons were conducted using the Wilcoxon rank-sum test implemented in ALDEx2, and *p*-values were adjusted using the Benjamini–Hochberg procedure. Taxa with adjusted *p*-values (we.eBH) < 0.05 were considered significantly differentially abundant. Venn diagrams were generated to identify shared and unique taxa among treatments, considering only taxa with relative abundances greater than 1%.

## 3. Results

### 3.1. Stability of Chlorella Population in the Biofloc System

A multifactorial ANOVA revealed significant differences related to both treatments and sampling time on chlorophyll concentration, with a greater accumulation in the treatments inoculated with *Chlorella* sp. (Table S2).

**Figure 2:**
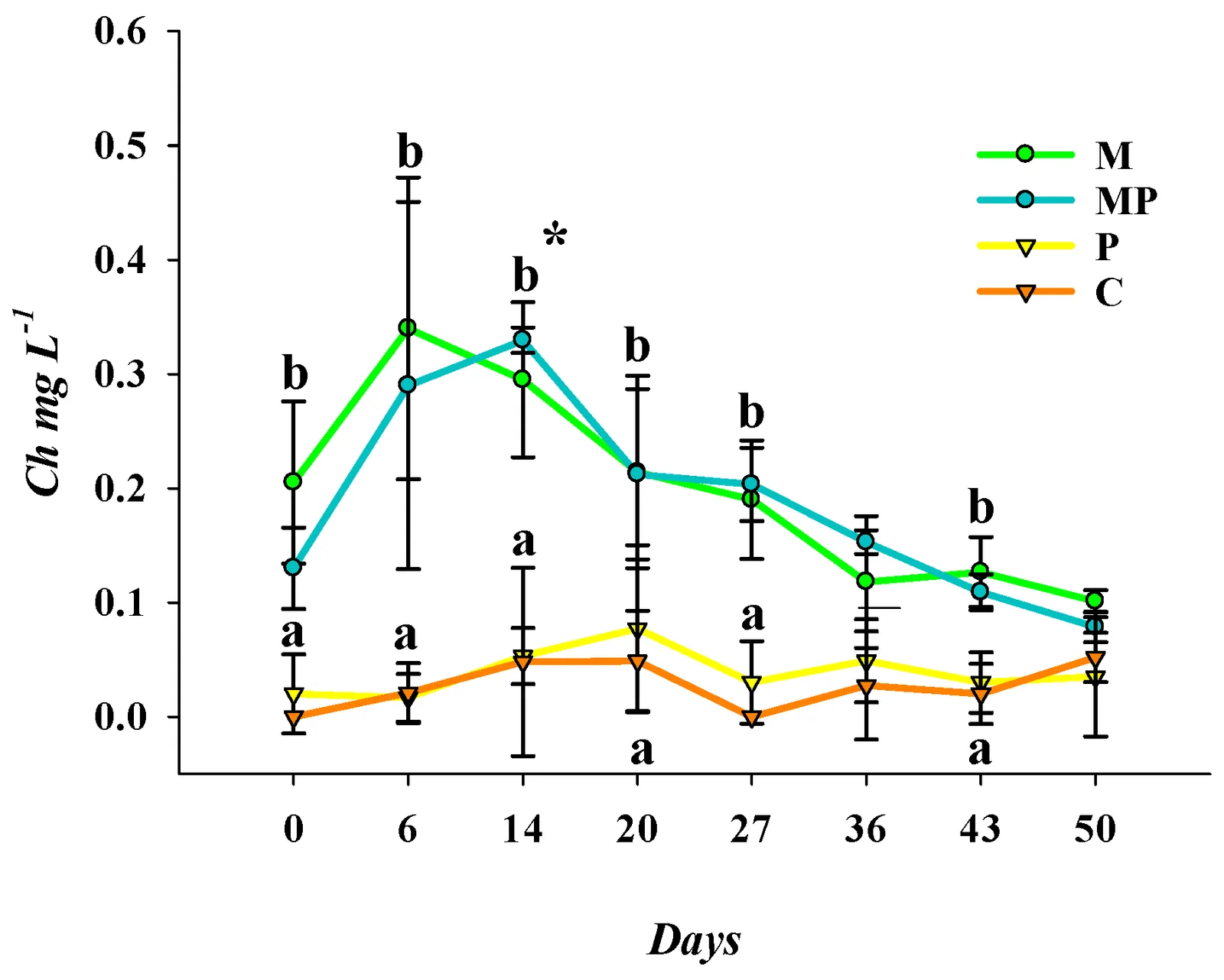
Total chlorophyll concentration through the experimental period. Different letters indicate significant differences between treatments (*p* < 0.05). The asterisk (*) indicates significant differences over time within the *Chlorella* sp. treatments (*p* < 0.05). The means comparison test used was Student–Newman–Keuls.

The temporal dynamics of chlorophyll concentration during the experimental period showed that the treatments inoculated with *Chlorella* sp. exhibited higher chlorophyll concentrations. However, although chlorophyll levels progressively declined after 14 days following inoculation, chlorophyll concentrations remained significantly higher than in the other treatments until day 43 (Figure 2).

### 3.2. Effect of Probiotic or Microalgae Addition on Water Quality

**Figure 3:**
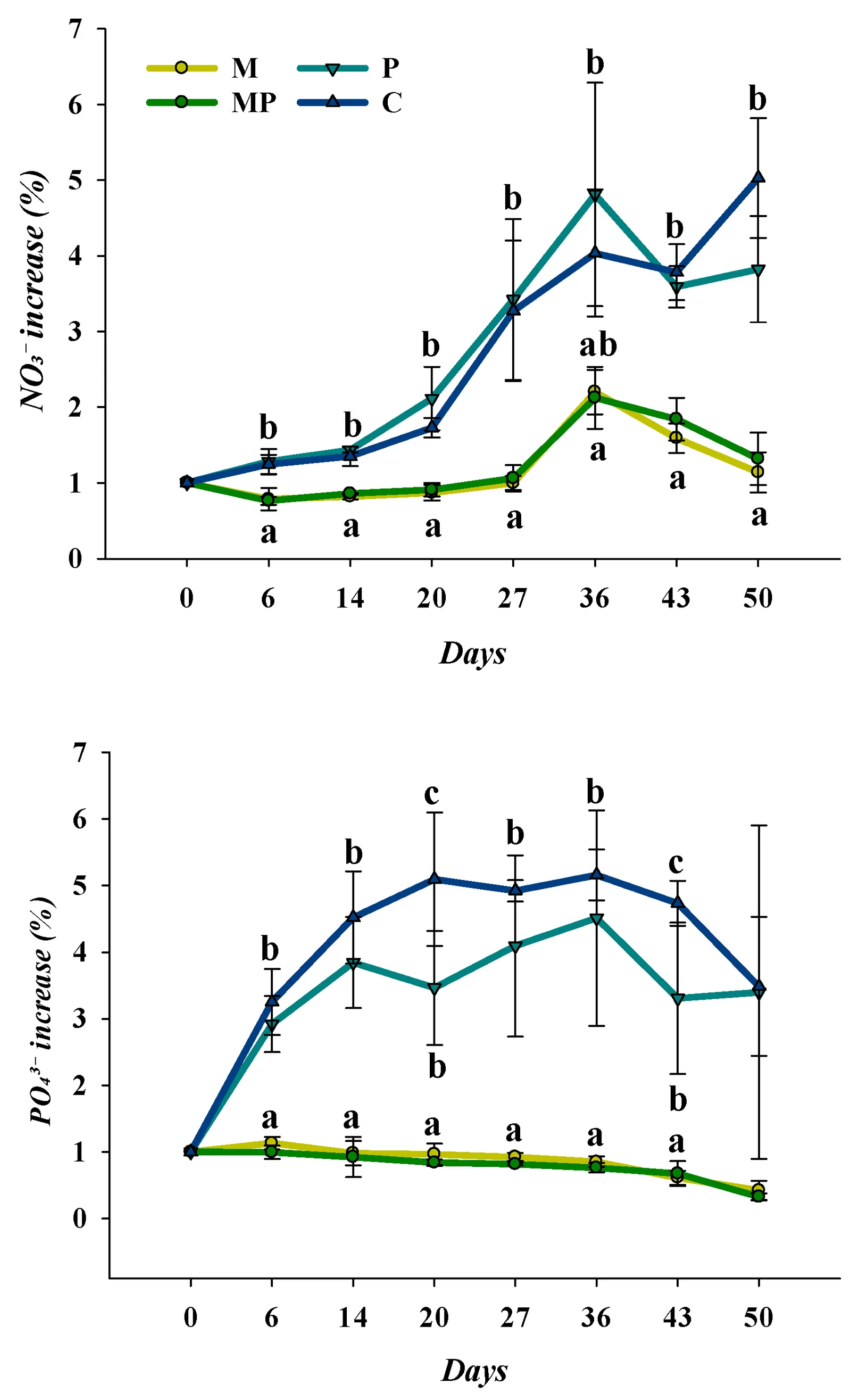
Nitrate and phosphate accumulation in the water over time. Values represented as mean ± standard error of the mean (*n* = 3). Different letters indicate significant differences between treatments (*p* < 0.05). The means comparison test used was Student–Newman–Keuls.

Water physicochemical parameters were maintained throughout the experimental period, at 27.6 ± 1.3 °C, salinity of 21.5 ± 4.3 PSU, alkalinity of 141 ± 50 mg L^−1^, pH 8.0 ± 1.5 and dissolved oxygen (DO) level 6.2 ± 1.3 mg L^−1^. No significant differences were observed in total suspended solids (TSS) among treatments (Table S3). Ammonia and nitrite remained within optimal ranges throughout the experimental period. Although nitrate and phosphate accumulated over time in all treatments, the increase relative to the initial concentration was markedly lower in the microalgae treatments (Figure 3). Despite this lower accumulation, the mean nitrate and phosphate concentrations remained significantly higher in these treatments, likely reflecting their higher initial concentrations rather than greater nutrient accumulation during the experimental period (Table S3).

### 3.3. Effect of Probiotic or Microalgae Addition on Nutritional Value of Biofloc

The presence of *Chlorella* sp. alone significantly reduced the crude protein content of the biofloc, whereas probiotic supplementation did not modify this parameter relative to the control. Despite these differences in protein content, no significant differences were detected in the energy content of the biofloc (Table 1).

**Figure 4:**
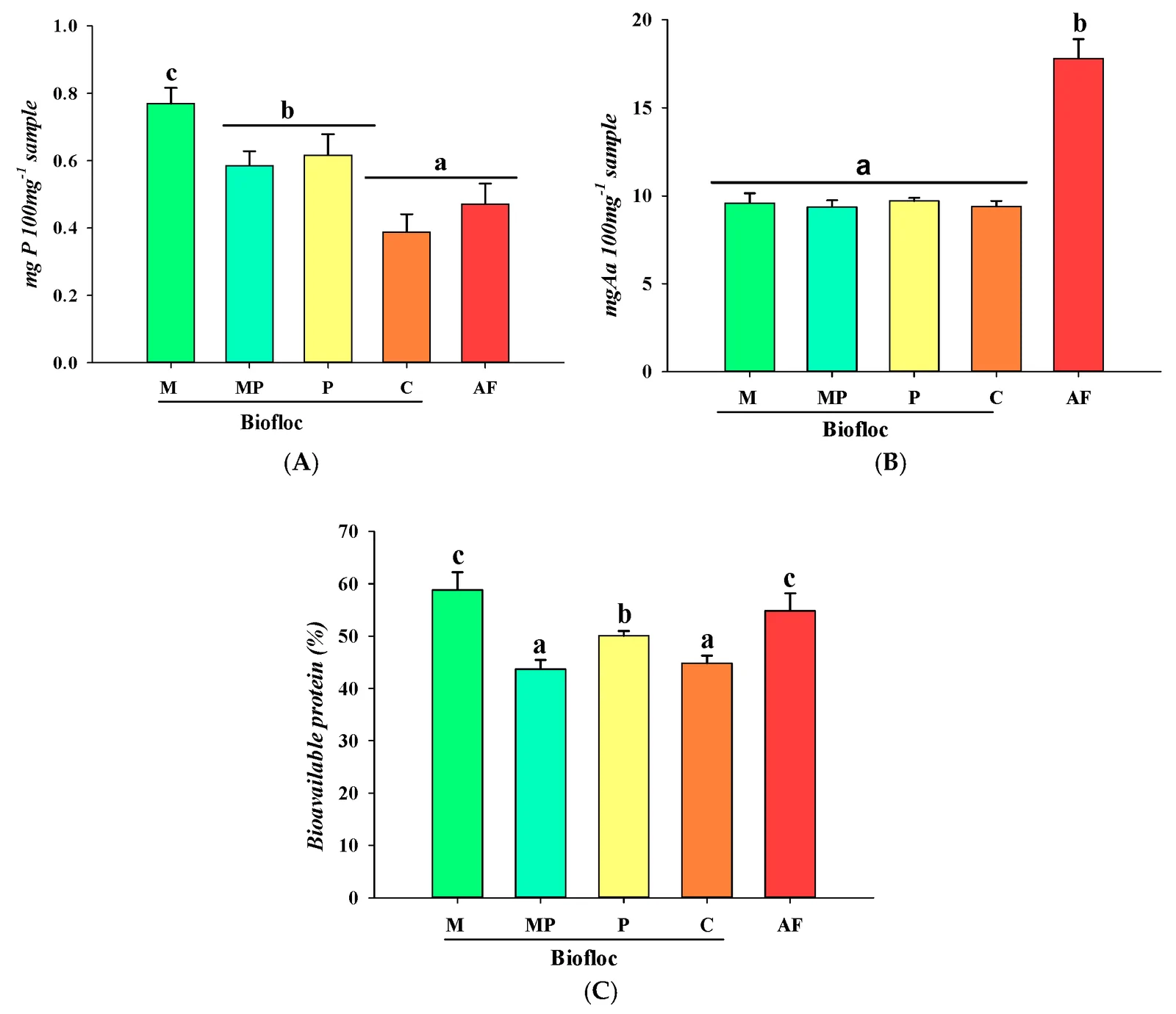
Biofloc bioavailability of phosphorus (mg phosphorus for 100 mg of sample) (**A**), nitrogen (mg amino acids for 100 mg of sample) (**B**) and protein (%; (mg of amino acids in sample/initial protein in sample) × 100) (**C**). Different letters indicate significant differences with a *p* < 0.05. AF (Artificial feed).

The potential bioavailability of nutrients present either in biofloc or artificial feed after simulated digestion by shrimp enzymes revealed a significantly higher release of bioavailable P from biofloc containing either microalgae or probiotics, compared to the values measured in control or the artificial feed. In addition, all biofloc samples presented similar values of nitrogen bioavailability (expressed as release of amino acids after enzyme hydrolysis) being significantly lower than that measured in the feed. When the values were corrected and expressed in relation to the initial protein contents present in samples, some differences arise and then biofloc containing microalgae showed a similar value to that measured for the feed, while the rest of biofloc showed significantly lower values (Figure 4A–C).

### 3.4. Effect of Probiotic or Microalgae Addition on Biofloc Microbial Community

**Figure 5:**
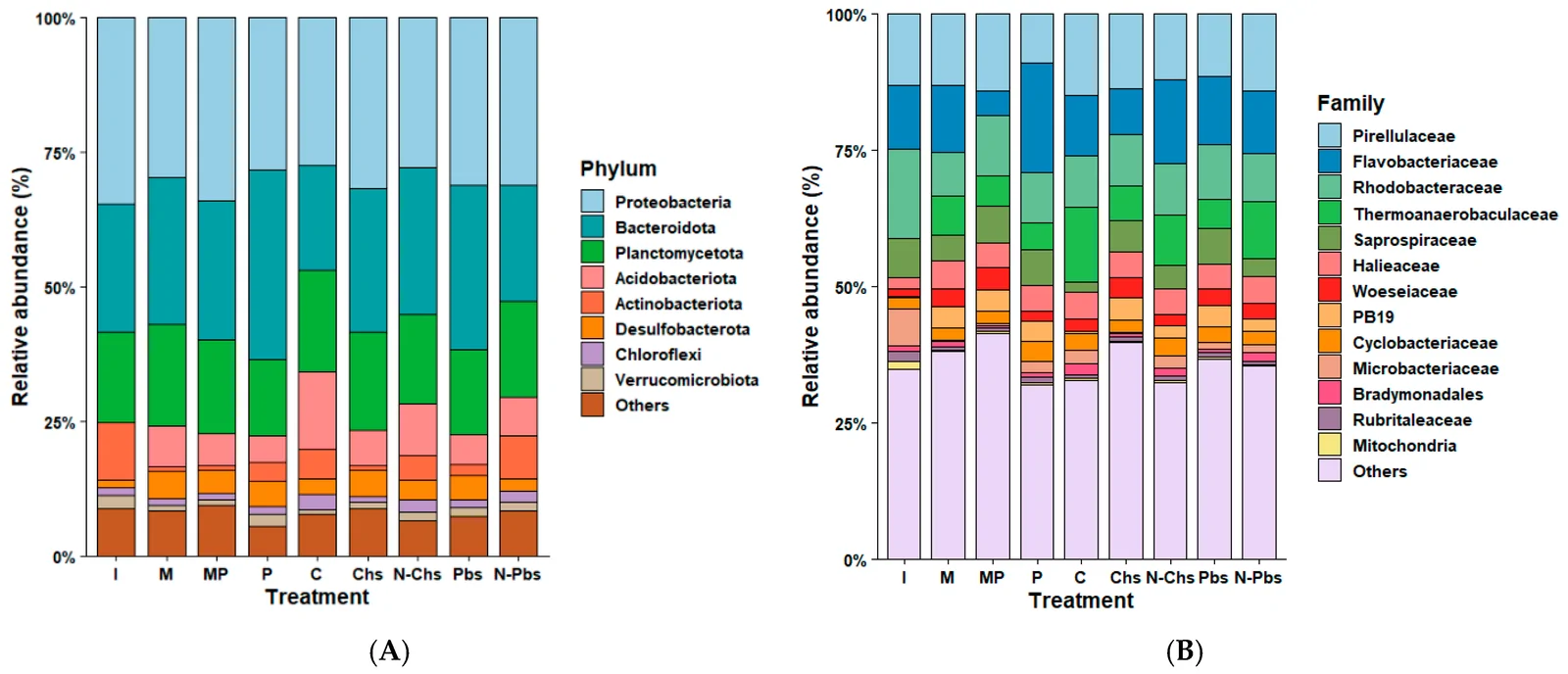
Relative abundance (%) of the bacterial community of biofloc. Phylum level (**A**); family level (**B**). Initials (I), treatments with *Chlorella* (Chs: M + MP) and without *Chlorella* (N-Chs: P + C), treatments with probiotics (Pbs: MP + P) and without probiotics (N-Pbs: M + C).

Metabarcoding analysis of the 16S rRNA gene from biofloc samples yielded an average of 60,326 input reads per sample. After quality filtering, 93.6% of the reads passed the filtering step, resulting in an average of 54,211 denoised reads per sample. Of these, approximately 89.8% corresponded to non-chimeric reads, which were retained for downstream taxonomic analysis across all treatments (Table S4). The metataxonomic analysis revealed the presence of 14 phyla, 58 classes, 115 orders, 185 families, and 298 genera. The bacterial community of the biofloc was primarily composed of the phyla Proteobacteria (29.87 ± 4.60%), Bacteroidota (26.01 ± 8.90%), Planctomycetota (17.31 ± 2.90%), and Actinobacteria (2.63 ± 5.47%), which together accounted for 75.82% of the total bacterial abundance (Figure 5A). At the family level, the bacterial community was dominated by Pirellulaceae (12.99 ± 3.16%), Flavobacteriaceae (11.88 ± 6.79%), Rhodobacteraceae (9.42 ± 2.05%), Saprospiraceae (6.08 ± 3.02%), Halieaceae (4.81 ± 1.65%), Microbacteriaceae (3.98 ± 3.94%), and Thermoanaerobaculaceae (3.92 ± 4.92%), which together accounted for 53.08% of the total bacterial abundance (Figure 5B).

Biofloc maturation altered the abundance of Acidobacteriota, which increased over time regardless of the treatment applied (Table 2). No significant differences among experimental groups were detected at any taxonomic level. However, when biofloc communities were analyzed according to the presence or absence of microalgae and bacterial probiotics, shifts in bacterial community composition were revealed. Actinobacteria displayed a significantly higher relative abundance in treatments lacking microalgae.

**Figure 6:**
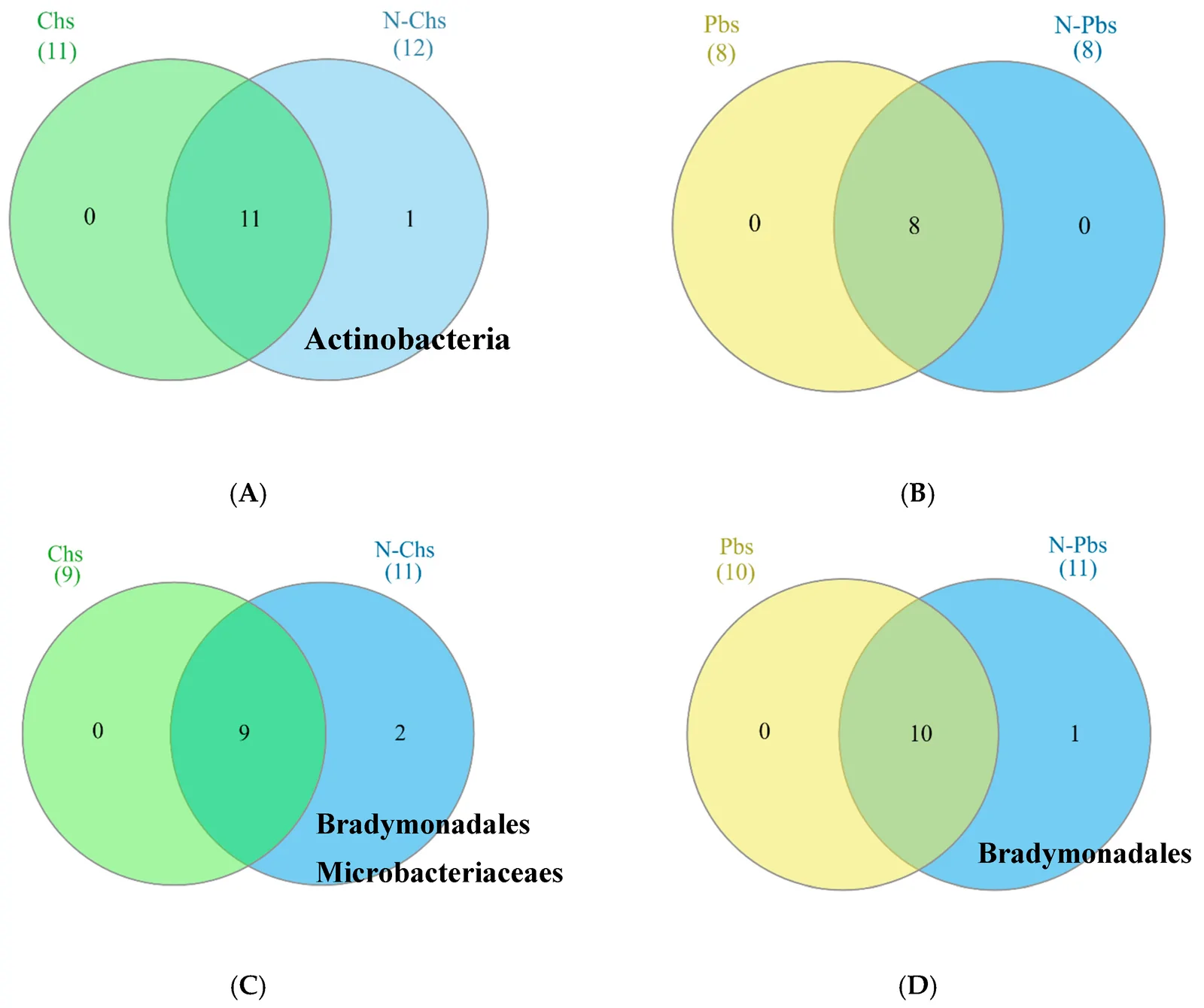
Venn diagrams showing the shared and unique bacterial taxa among the experimental treatments in biofloc samples. Diagrams (**A**,**B**) represent comparisons at the phylum level, whereas diagrams (**C**,**D**) represent comparisons at the family level. Numbers indicate the taxa exclusively detected in each treatment and those shared among two or more treatments. Treatments with *Chlorella* (Chs: M + MP) and without *Chlorella* (N-Chs: P + C); treatments with probiotics (Pbs: MP + P) and without probiotics (N-Pbs: M + C).

This pattern was also reflected in the Venn diagram (Figure 6A), where Actinobacteria were the only phylum exclusively detected in treatments without microalgae. In contrast, the presence of *Chlorella* sp. influenced the bacterial community structure by significantly decreasing the relative abundance of the family Cyclobacteriaceae (Table 2). Finally, the absence of probiotic was associated with a significant reduction in the relative abundance of Rhodobacteraceae (Table 2). The Venn diagram of principal families comparing treatments with and without *Chlorella* sp. (Figure 6C) shows that Bradymonadales and Microbacteriaceae did not exceed 1% relative abundance in the microalgae treatments and, therefore, were not considered shared between the two groups. Similarly, the Venn diagram comparing treatments with and without probiotic addition (Figure 6D) indicates that Bradymonadales was present only in the treatments without bacterial probiotics (>1%).

In terms of bacterial biodiversity, the only significant differences observed were the increases in the Shannon and Simpson alpha diversity indices when comparing the initial and final sampling points in the control group at the phylum level (Table S4).

**Figure 7:**
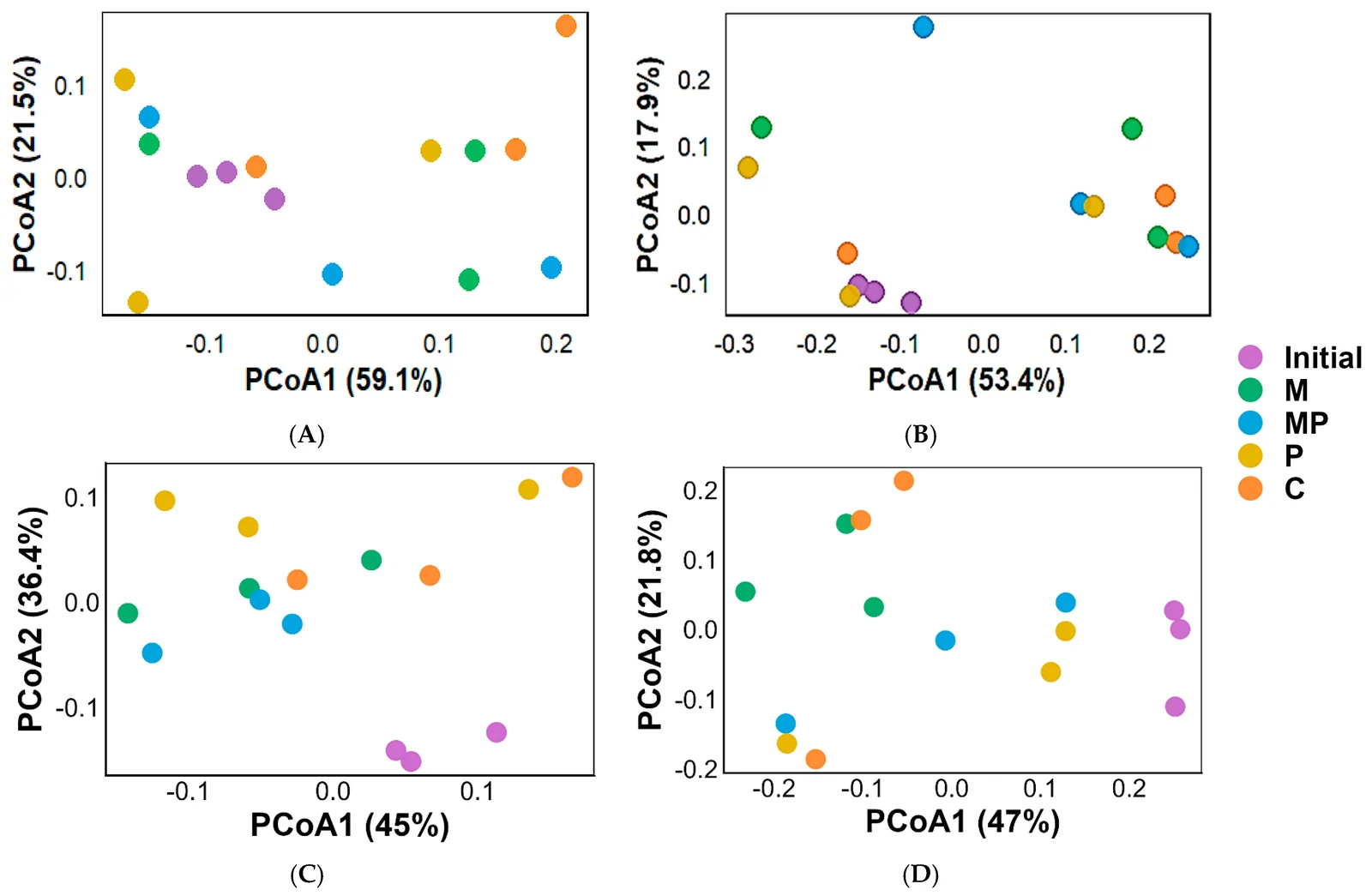
Principal coordinates analysis (PCoA) based on Bray–Curtis dissimilarities showing the beta diversity of bacterial communities in biofloc and shrimp intestine samples (*n* = 3; 2 shrimp per tank). Biofloc samples at the phylum level, including initial and final sampling points (**A**); biofloc samples at the family level, including initial and final sampling points (**B**); shrimp intestine samples at the phylum level, including initial and final sampling points (**C**); and shrimp intestine samples at the family level, including initial and final sampling points (**D**). Each point represents an individual sample, and distances between points reflect differences in bacterial community composition.

In addition, the PCoA based on Bray–Curtis dissimilarities showed a strong overlap among treatments in initial and final biofloc samples at both the phylum (Figure 7A) and family (Figure 7B) levels, with no clear clustering pattern. These patterns were supported by PERMANOVA results, which revealed no significant differences in bacterial beta diversity among treatments, either when initial and final sampling points were analyzed together or when only final samples were considered (Table S7).

### 3.5. Effect of Probiotic or Microalgae Addition on Shrimp Performance

#### 3.5.1. Shrimp Growth and Nutritional Composition

Shrimp receiving microalgae supplementation exhibited significantly lower growth and survival rates than those from the probiotic and control treatments. Conversely, probiotic supplementation significantly improved most zootechnical performance parameters compared to microalgae treatments, but not to the control (Table 3).

The nutritional composition of shrimp was also affected by the treatments. The probiotic treatment exhibited the highest crude protein content, whereas no significant differences were observed for dry matter, lipid, ash, or energy content (Table 4).

#### 3.5.2. Intestinal Microbiota

**Figure 8:**
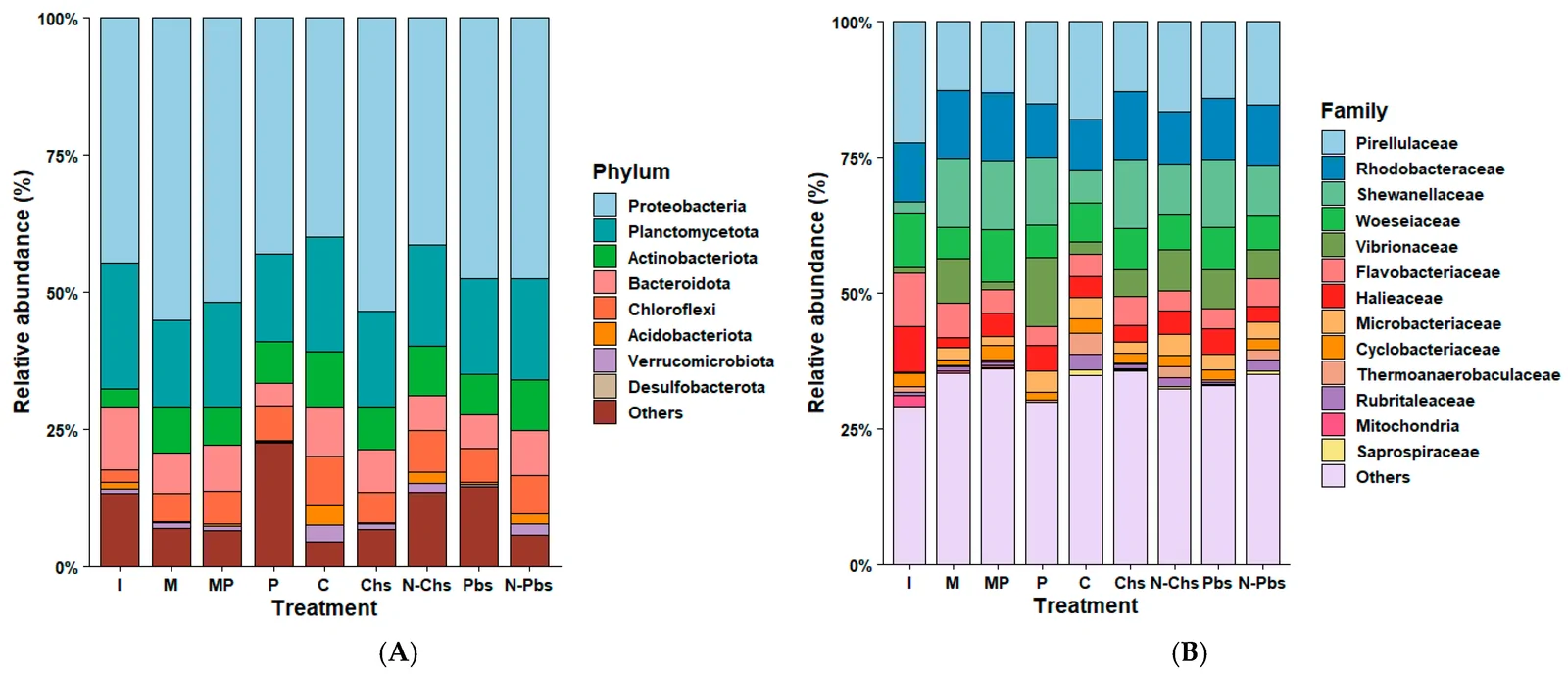
Relative abundance (%) of the bacterial community in intestines of shrimp. Phylum level (**A**); family level (**B**). Initials (I), treatments with *Chlorella* sp. (Chs: M + MP) and without *Chlorella* sp. (N-Chs: P + C); treatments with probiotics (Pbs: MP + P) and without probiotics (N-Pbs: M + C).

Metabarcoding analysis of the 16S rRNA gene from shrimp intestine samples yielded an average of 22,095 input reads per sample. Following quality filtering, 92.3% of the reads passed the filtering step, producing an average of 19,635 denoised reads per sample. Among these, 83.6% corresponded to non-chimeric reads, which were used for subsequent microbial community analyses (Table S4). The metataxonomic analysis revealed the presence of 23 phyla, 37 classes, 95 orders, 131 families, and 207 genera. In terms of relative abundance, the intestinal bacterial community was dominated by the phyla Proteobacteria (46.45 ± 6.36%), Planctomycetota (17.33 ± 3.10%), Actinobacteria (7.80 ± 2.50%), Bacteroidota (6.92 ± 2.77%), and Chloroflexi (6.29 ± 2.33%), which together accounted for 84.79% of the total bacterial abundance (Figure 8A). Similarly, at the family level, the intestinal microbiota was predominantly composed of Pirellulaceae (14.66 ± 3.40%), followed by Rhodobacteraceae (10.80 ± 1.50%), Shewanellaceae (9.02 ± 4.86%), Woeseiaceae (7.11 ± 1.83%) and Flavobacteriaceae (4.47 ± 2.33%) (Figure 8B).

As in the biofloc bacterial profile, the taxonomic relative abundance analysis at the phylum level revealed specific temporal changes (Table 5). Both Actinobacteria and Chloroflexi increased over time, whereas Acidobacteriota decreased in all groups except the intestine of shrimp control treatment. In contrast, only Acidobacteriota showed significant differences among treatments, with a significantly higher relative abundance in the intestinal microbiota of shrimp from the control treatment. Furthermore, when intestinal samples were grouped according to the presence or absence of microalgae or bacterial probiotics (Table 5), the inclusion of *Chlorella* sp. was associated with lower relative abundances of Acidobacteriota and Chloroflexi. Similarly, the presence of bacterial probiotics also resulted in a reduction in the relative abundance of Acidobacteriota.

At the family level, the families Halieaceae and Thermoanaerobaculaceae significantly decreased at the end of the experimental period in all groups, except in control groups for the Thermoanaerobaculaceae. In contrast, Microbacteriaceae showed an increase; meanwhile, Cyclobacteriaceae decreased in the microalgae group. These families were also altered by the experimental conditions. Halieaceae, Pirellulaceae, and Cyclobacteriaceae families showed significantly lower relative abundances in shrimp receiving only microalgae supplementation (M), whereas the highest abundances were observed in the control treatment. The MP and P treatments displayed intermediate abundance values. Conversely, both treatments containing *Chlorella* sp. resulted in a lesser relative abundance of Microbacteriaceae than probiotic and control groups. Finally, Thermoanaerobaculaceae showed its lowest abundance in shrimp from the M treatment, increased significantly in the MP and P treatments, and reached its highest abundance in the control group.

If the presence/absence of *Chlorella* sp. and probiotics are considered, the influence of both becomes more evident. As shown in Table 5, the absence of both microalgae and probiotic supplementation resulted in significantly higher relative abundances of the phylum Acidobacteriota and the family Thermoanaerobaculaceae. The effect of *Chlorella* sp. was also evident for the Microbacteriaceae family and Chloroflexi phylum, whose relative abundance decreased in the presence of the microalga. Similarly, probiotic supplementation led to a reduction in the relative abundance of Flavobacteriaceae in the shrimp intestinal microbiota.

Unlike the biofloc bacterial community, the intestinal microbiota was affected by the experimental conditions. According to the Chao1 richness index, bacterial diversity decreased in the control group over the course of the experiment (Table S6). Meanwhile, family-level analyses revealed a reduction in Chao1 richness between the initial and final sampling points, independently of the treatment (Table S5).

Bacterial beta diversity in the shrimp intestinal microbiota was assessed by PCoA based on Bray–Curtis dissimilarities at both the phylum (Figure 7C) and family (Figure 7D) levels. The ordination plots suggested a temporal shift in bacterial community composition between the initial and final sampling points, with no evident separation among treatments. Accordingly, PERMANOVA detected significant differences when analyzing initial and final samples but not among final treatments (Table S7).

**Figure 9:**
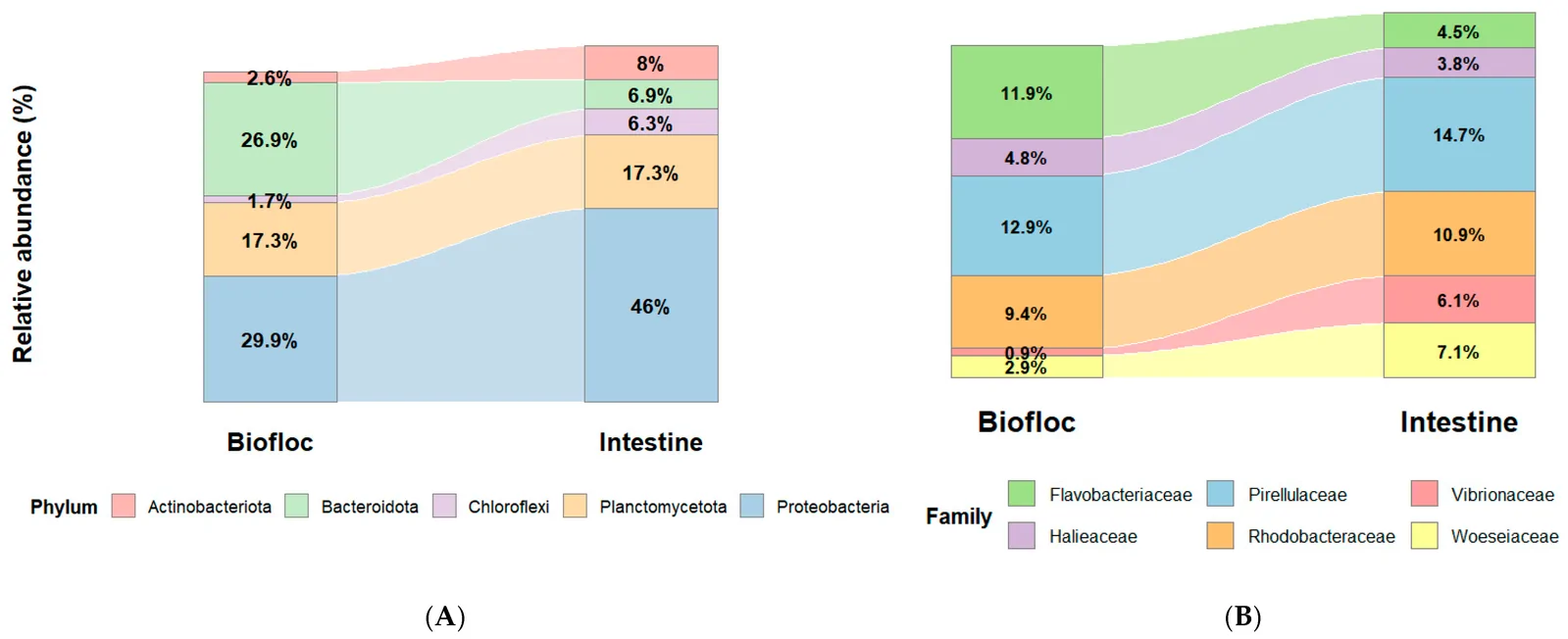
Sankey (alluvial) diagrams illustrating the relative abundance (RA, %) of the most representative bacterial taxa shared between biofloc and intestinal microbiota of *P. vannamei*. Dominant shared taxa at the phylum level (**A**). Dominant shared taxa at the family level (**B**). The width of each flow is proportional to the relative abundance of the corresponding taxon, highlighting its contribution to the microbial communities detected in both biofloc and shrimp intestines.

When evaluating the potential interaction between the biofloc and intestinal microbiota, the dominant bacterial phyla were found to be shared between both microbial communities, although their relative abundances differed (Figure 9A). Proteobacteria was the most abundant shared phylum, increasing from 29.9% in biofloc to 46.0% in the intestine. Planctomycetota showed similar abundances in both compartments (17.3%), whereas Bacteroidota decreased from 26.9% to 6.9%. In contrast, Actinobacteria and Chloroflexi were enriched in the intestinal microbiota, increasing from 2.6% to 8.0% and from 1.7% to 6.3%, respectively. At the family level (Figure 9B), Pirellulaceae was the dominant shared family, increasing from 12.9% in biofloc to 14.7% in the intestine. Rhodobacteraceae remained relatively stable (9.4–10.9%), whereas Flavobacteriaceae decreased from 11.9% to 4.5%. Conversely, Woeseiaceae and Vibrionaceae were more abundant in the intestine, increasing from 2.9% to 7.1% and from 0.9% to 6.1%, respectively. Overall, the Sankey diagrams highlighted substantial differences in the relative abundance of shared bacterial taxa between biofloc and intestinal microbiota.

## 4. Discussion

Unlike previous studies based on continuous microalgae addition [3,26,46,47], the present study demonstrated that a single inoculation was sufficient to maintain a stable population of *Chlorella* sp. within the biofloc microbial community for more than 40 days. This persistence exceeded the 32-day period previously reported by our research group under similar experimental conditions [25]. A possible explanation is that *Chlorella* sp. is capable of establishing mutualistic associations with bacteria that stimulate algal growth while simultaneously benefiting from algal organic exudates [20]. Previously, the 86% of the symbiotic bacteria exerted a positive effect on *Chlorella* growth, highlighting the potential role of bacterial symbiosis in enhancing algal performance [48]. In addition, previous studies have also described mutualistic interactions between *C. vulgaris* and *Pseudomonas alcaligenes* [49], as well as with *Bacillus licheniformis* [50]. However, these interactions are highly dynamic and may shift from mutualism to competition as microbial biomass accumulates. Under such conditions, increased competition for nutrients and ecological niches, together with enhanced turbidity and self-shading caused by the accumulation of both bacterial and algal biomass, may reduce light availability and ultimately limit microalgal persistence [20,51].

Because light availability is a key factor regulating *Chlorella* establishment and persistence in biofloc systems, the experimental design should be considered when interpreting these findings. In the present study, the *Chlorella*-supplemented treatments were maintained under a 12 L:12 D photoperiod, whereas the non-supplemented treatments were kept in darkness. This approach was adopted because previous work demonstrated that artificial illumination is required to establish and maintain a stable *Chlorella* population in biofloc systems [25]. Nevertheless, this design does not allow the effects of photoperiod to be fully separated from those of *Chlorella* supplementation. Therefore, future studies should include an illuminated control without microalgal supplementation to distinguish the specific effects of light exposure from those of *Chlorella* addition.

### 4.1. Effects of Microalgae and Probiotics on Water Quality and Nutrient Removal

The successful establishment of *Chlorella* sp. was accompanied by measurable effects on nutrient dynamics within the biofloc system. The lower accumulation of nitrate and phosphate observed in the microalgae treatments confirms the bioremediation potential previously reported for *Chlorella*-based and microalgae-based biofloc systems [25,52,53]. The ability of phytoplankton to incorporate nitrogen and phosphorus to support growth is well established [54,55]. For example, one study showed that the addition of *Platymonas* sp. (1 × 10^5^ cell⋅mL^−1^) every 4 days significantly reduced nitrite-N accumulation in 50% of biofloc environments [27]. Likewise, the co-culture of *Nannochloropsis oculata* with *P. vannamei*, achieved through a single initial inoculation of approximately 5 × 10^11^ algal cells, resulted in significantly lower phosphate, ammonium, and nitrite concentrations compared with control groups [56]. Furthermore, other studies maintained *Thalassiosira pseudonana* and *N. oculata* at densities of 1 × 10^5^ cells mL^−1^ through semi-continuous additions every two days and observed significantly lower concentrations of TAN, nitrite-N, nitrate and orthophosphate-P [53]. Collectively, these studies indicate that microalgal supplementation effectively limits the accumulation of dissolved inorganic nitrogen and phosphorus compounds, thereby improving water quality and promoting a more stable culture environment.

Probiotic supplementation did not significantly reduce nitrate or phosphorus accumulation compared with the control treatment in the present study. Our results are consistent with similar studies who evaluated several commercial probiotic formulations dominated by *Bacillus* spp. in mature biofloc systems and found no significant changes in TAN, nitrite-N, or nitrate-N concentrations despite an increase in the abundance of ammonia-oxidizing bacteria [57]. Finally, the combined treatment improved water quality in comparison to both the probiotic-only and control treatments, but did not clearly outperform the microalgae-only treatment (M). Considering that probiotic supplementation alone did not significantly reduce nitrate or phosphorus concentrations, whereas the presence of *Chlorella* sp. was associated with lower accumulation of these nutrients, it is likely that the improvement observed in the combined treatment was driven primarily by the activity of the microalga.

### 4.2. Effect of Probiotic and/or Microalgae Addition on Biofloc Nutritional Composition

The nutritional composition of the biofloc obtained in the present study was broadly comparable to that reported for molasses-based BFT systems. Protein and lipid contents fell within the ranges commonly described for biofloc biomass, indicating a nutritional quality similar to that observed in previous studies [54,55,56]. In contrast, ash content was relatively high compared with most freshwater biofloc systems, although similar values have been reported under marine conditions [58]. This elevated mineral fraction likely reflects the influence of salinity on biofloc composition and the accumulation of inorganic material within the aggregates.

Furthermore, higher crude protein levels were recorded in the dark-maintained treatments (P and C), likely due to the predominance of heterotrophic microbial populations, which are characterized by a high capacity for nitrogen assimilation and the production of protein-rich microbial biomass [1,59]. In contrast, the M treatment showed the lowest protein content, possibly as a consequence of a greater contribution of autotrophic populations [59]. However, the continuous addition of probiotic bacteria increased the protein content in the MP treatment, reaching values similar to those observed in the dark-maintained treatments.

Nevertheless, the potential digestive bioavailability of the protein fraction for shrimp evidenced differences being significantly higher when *Chlorella* sp. was present. This finding suggests that protein assimilation efficiency may not be determined solely by total protein concentration, but also by the diversity of protein sources present within the biofloc matrix, including photoautotrophic, bacterial, and zooplanktonic components [60,61]. In fact, previous studies reported that the addition of *Chlorella* sp. increased zooplankton biodiversity [25], which may enhance amino acid release and absorption, thereby improving overall nutritional utilization. Although *Chlorella* sp. have structural features that may limit direct protein accessibility, the microalga displays a high nitrogen release rate (approximately 40%), in contrast to bacteria (~2 μm), which typically release 15–26% nitrogen [62]. The higher protein bioavailability observed in the present study may not be exclusively related to the direct contribution of *Chlorella* sp. biomass, but also to an indirect trophic effect mediated by the zooplankton community. Consequently, the greater availability of live feed organisms could have enhanced the transfer of protein-rich biomass through the food web, providing a more bioaccessible protein source for shrimp compared to the direct consumption of biofloc particles alone.

Probiotic supplementation also resulted in relatively high protein bioavailability, which may be associated with the stimulation of digestive enzyme activity previously reported for biofloc-derived probiotic bacteria [63]. However, the combined treatment did not further improve protein bioavailability, suggesting that the mechanisms responsible for enhanced digestibility in each treatment were not additive. To our knowledge, this is the first study evaluating biofloc protein bioavailability using an in vitro digestion assay. Although direct comparisons are limited, the relatively high values obtained contrast with the low apparent digestibility coefficients previously reported for biofloc meals in *P. vannamei* [58], supporting the potential nutritional value of fresh biofloc-associated protein.

In addition to protein utilization, the presence of *Chlorella* sp. and probiotics improved phosphorus bioavailability in the biofloc. This effect is likely associated with the ability of *Chlorella* sp. [64,65] and *Bacillus* spp. [66,67] to assimilate inorganic phosphorus from the surrounding environment. Given the importance of phosphorus for exoskeleton formation, osmoregulation and growth in shrimp [68], this enrichment may be particularly relevant under current aquaculture conditions, where the progressive replacement of fishmeal has reduced phosphorus availability in commercial feeds [69].

### 4.3. Effect of Probiotic and/or Microalgae Addition on Biofloc Microbial Composition

The microbial community is a key functional component of biofloc systems, contributing to nutrient transformation, organic matter recycling, and overall system stability [9]. In our study, only the control biofloc showed a progressive increase in bacterial alpha diversity over time, whereas the addition of *Chlorella* sp., the probiotic, or both microorganisms in combination appeared to constrain this natural diversification process. Nevertheless, beta diversity analyses revealed a high degree of similarity among treatments, indicating that the overall bacterial community structure remained relatively stable and that treatment effects were mainly restricted to specific bacterial taxa.

However, at the taxonomic level, the presence of *Chlorella* sp. was associated with a reduction in the relative abundance of Actinobacteria, particularly the family Microbacteriaceae, as well as Cyclobacteriaceae. Both families have been identified as core members of biofloc microbial communities and are closely related to nutrient recycling and nitrogen assimilation processes [70,71]. Members of Cyclobacteriaceae frequently coexist with bacterial consortia involved in ammonium removal in saline lakes and wastewater treatment systems, benefiting from the organic compounds generated during these processes [72]. Similarly, fluctuations in Microbacteriaceae abundance have been shown to closely track dissolved ammonium concentrations in aquatic ecosystems, due to their metabolic assimilation [73]. Therefore, the decrease in both bacterial groups observed in the presence of *Chlorella* may be related to competition for ammonium. Since ammonium is the preferred nitrogen source for *Chlorella* growth, microalgal uptake may have reduced its availability for ammonium-associated bacterial taxa.

Significant modifications in the biofloc bacterial composition following the addition of *Platymonas* sp. have been reported [27]. Similarly, the incorporation of *Nannochloropsis oculata* and *Thalassiosira weissflogii*, either individually or in combination, altered the bacterial community of shrimp culture water [74].

On the other hand, probiotic supplementation also modified the bacterial community structure. Treatments receiving the probiotic showed a higher relative abundance of Rhodobacteraceae together with the exclusive presence of the order Bradymonadales (>1%). Rhodobacteraceae is considered one of the most abundant and metabolically versatile bacterial families in biofloc systems, participating in organic matter degradation, ammonium assimilation, and denitrification processes [70]. Furthermore, several genera within this family are capable of heterotrophic nitrification, oxidizing ammonium to nitrite or nitrate while simultaneously utilizing organic carbon as an energy source [71]. This metabolic strategy is particularly advantageous in biofloc systems, which are characterized by high concentrations of dissolved organic matter and where conventional autotrophic nitrifiers may be limited [75]. Consequently, the increase in Rhodobacteraceae observed in the presence of probiotic may reflect an indirect selection of bacterial groups involved in nitrogen transformation. Likewise, the exclusive detection of Bradymonadales in probiotic treatments suggests that probiotic supplementation promoted the establishment of specific bacterial taxa. Previous studies demonstrated that Bradymonabacteria possess a broad-spectrum predatory capacity, enabling them to invade and efficiently eliminate neighboring bacterial colonies [76]. Such a predatory lifestyle provides a strong ecological advantage in aquatic ecosystems and coastal sediments. Finally, Acidobacteriota increased in relative abundance over time in all treatments, including the control. This consistent pattern suggests that the proliferation of this phylum was primarily associated with the natural maturation of the biofloc community rather than with the specific effects of microalgal or probiotic supplementation [77].

### 4.4. Effect of Probiotic and/or Microalgae Addition on Shrimp Productivity

The zootechnical parameters evaluated revealed a differential effect of the treatments on shrimp performance. Overall, the values obtained in treatments P and C were within the ranges commonly reported for *P. vannamei* cultured in biofloc systems. Studies conducted over culture periods ranging from 30 to 70 days have reported survival rates between 70 and 91%, specific growth rates (SGRs) ranging from 1.35 to 2.82% day^−1^, feed conversion ratios (FCRs) between 1.3 and 1.9, and protein efficiency ratios (PERs) close to 2.0 [78,79,80,81]. In the present study, treatment P achieved a survival rate of 77.9%, an SGR of 2.2% day^−1^, an FCR of 1.3, and a PER of 2.0, placing it within the upper range previously reported for biofloc systems.

The use of probiotics in biofloc systems is a widespread practice, and numerous studies have reported improvements in growth, survival, and feed utilization in *P. vannamei* following the incorporation of different bacterial strains [8,82,83]. For instance, *B. licheniformis* improved growth and feed utilization [82], and *B. subtilis* enhanced both growth and survival [83]. Similarly, one study reported that the application of multistrain probiotic consortia, including *Bacillus* spp., significantly increased shrimp growth and survival while reducing FCR from 2.7 to 1.4 [84]. Furthermore, the PER recorded in this treatment was almost identical to that previously reported for shrimp cultured in biofloc systems (1.99) [80], suggesting an efficient utilization of dietary protein. Overall, the probiotic and control treatments exhibited comparable zootechnical performance, indicating that probiotic supplementation did not provide a clear productive advantage over the control under the experimental conditions of this study. Nevertheless, both treatments outperformed the *Chlorella* sp.-supplemented groups, which consistently showed lower survival and growth rates.

The lack of a positive response to microalgal supplementation may be associated with the elevated nitrate concentrations recorded throughout the culture period. In the present study, the high nitrate and phosphate concentrations detected in the microalgae treatments were most likely related to the inoculation procedure. *Chlorella* sp. was produced through successive culture scale-up using nutrient-enriched culture media [85,86]. Harvesting microalgal biomass by centrifugation or washing is widely recognized as an energy-intensive and costly downstream process, particularly at large culture volumes [85,87]. Therefore, the entire *Chlorella* culture, including the residual culture medium, was directly inoculated into the experimental tanks. In addition, water samples were collected immediately after inoculation, and the first measurements already showed high nitrate concentrations in the microalgae treatments, indicating that nutrient carry-over associated with the microalgal inoculum was the most plausible explanation for these elevated concentrations. Although *Chlorella* subsequently assimilated part of these nutrients, reducing their accumulation over time, this bioremediation effect was insufficient to compensate for the high initial nutrient input.

For this reason, although nitrate toxicity in *P. vannamei* is not yet fully understood, several studies have demonstrated negative effects on growth and survival when nitrate concentrations exceed certain thresholds. Juvenile *P. vannamei* reared in biofloc systems at a salinity of 25 g L^−1^ and exposed to nitrate concentrations of 139.45 and 278.91 mg L^−1^ for 10–30 days showed significantly reduced growth [88]. Likewise, the sensitivity of juvenile *P. vannamei* to nitrate has been shown to depend strongly on salinity, with proposed safety levels of 45.3 and 90.0 mg L^−1^ at salinities of 1 and 3 g L^−1^, respectively [89], and 60.05 and 127.61 mg L^−1^ at salinities of 5 and 10 g L^−1^, respectively [90]. Moreover, nitrate concentrations above 220–300 mg L^−1^ have previously been associated with reduced growth and survival [90]. Other crustaceans, like juveniles of *M. rosenbergii* exposed to 45.6 and 91.2 mg L^−1^ nitrate for 40 days, exhibited significant reductions in both growth and survival [91]. In the present study, shrimp in the microalgae treatments were exposed from the beginning of the experiment to nitrate concentrations exceeding 235 mg L^−1^, values above those previously associated with reduced shrimp performance. Therefore, it is possible that the elevated nitrate concentrations introduced with the inoculum imposed physiological stress on the cultured shrimp. Consequently, it cannot be ruled out that this nitrate exposure contributed to the lower growth and survival observed in the microalgae treatments, potentially masking some of the beneficial effects associated with *Chlorella* supplementation.

These findings highlight the importance of optimizing the microalgal inoculation strategy to minimize nutrient carry-over from the culture medium, which may confound the evaluation of the biological effects of the microalgae. One possible approach would be to separate biomass from the culture medium by centrifugation prior to inoculation, as this method provides high recovery efficiency while maintaining cell viability, although its high energy demand limits its applicability at aquaculture production [92]. Other alternatives, such as filtration, flocculation, or flotation, could also be considered, although they present limitations related to recovery efficiency, the use of chemical additives, or their applicability to small microalgae such as *Chlorella* [92,93,94]. Alternatively, the biofloc system could be established from the outset through the simultaneous inoculation of microalgae and bacteria, potentially reducing the volume of microalgal inoculum required and the associated nutrient input. Indeed, the simultaneous incorporation of *Chlorella pyrenoidosa* and bacteria during biofloc establishment has been shown to promote biofloc formation, with the initial microalgal concentration being a key factor determining its subsequent development [95].

On the other hand, the higher crude protein content observed in shrimp supplemented with probiotics (Ps) may be related to improved nutrient utilization and protein deposition promoted by the probiotic strains *Bacillus subtilis* and *Bacillus licheniformis*. Similar results have been reported in biofloc systems, where the application of these bacterial species increased shrimp crude protein content while simultaneously improving growth performance and feed conversion efficiency [96].

On the other hand, although no evidence of cannibalism was observed during the experimental period, the potential density-compensation effect cannot be definitively excluded, as stocking density was not included as a covariate in the statistical analysis.

### 4.5. Effect of Probiotic and/or Microalgae Addition on Intestinal Microbial Composition

It has been demonstrated that the microbial community associated with biofloc can positively influence the intestinal microbiota, growth performance, and innate immune response of shrimp [70]. In the present study, Proteobacteria was the dominant phylum in the intestinal microbiota of *P. vannamei*, consistent with previous reports under biofloc culture conditions [70,97,98,99,100,101,102]. Within this phylum, Rhodobacteraceae was among the most abundant families and includes bacterial groups associated with antimicrobial activity and immune modulation [98,103]. Planctomycetota was also highly abundant, occurring at levels comparable to those observed in the biofloc and likely contributing to the degradation of complex polysaccharides [104]. Likewise, the presence of Bacteroidota, Actinobacteria, Chloroflexi, and Acidobacteriota suggests an important role of the intestinal microbiota in organic matter degradation and nutrient recycling [99,105,106,107]. At the family level, alpha diversity analysis revealed a significant decrease in the Chao1 richness index, following a common pattern across all experimental groups. Previous studies have reported that probiotic supplementation can modify the beta diversity of the intestinal microbiota in biofloc systems [108]. However, under the experimental conditions of the present study, no significant differences among treatments were detected, suggesting that the effects of probiotic and/or *Chlorella* supplementation on the overall bacterial beta diversity were limited compared with the temporal changes occurring during the culture period. Regardless of treatment, the intestinal microbiota underwent temporal changes throughout the culture period, particularly the increase in Actinobacteria and Chloroflexi, suggesting an increased importance of nutrient-recycling functions within the intestinal microbiota during culture development [106]. A contrasting pattern was observed for Acidobacteriota. While its relative abundance increased in the control treatment, it decreased in all supplemented groups. Consequently, Acidobacteriota was also the only phylum showing significant differences in relative abundance among treatments at the end of the experiment. Although this group has been associated with the degradation of recalcitrant compounds and organic matter mineralization [106], and chitinolytic species have been reported within this phylum [109], its ecological role in the crustacean intestine remains poorly understood. Nevertheless, other studies demonstrated that supplementation with *B. subtilis* reduced the abundance of Acidobacteriota in the shrimp intestine, consistent with the decrease observed in the probiotic-containing treatments [110]. Likewise, the reduction observed in the *Chlorella* sp. groups suggests that the microalga also exerted selective pressure on this bacterial group, possibly through modifications in nutrient availability or indirect alterations of the intestinal microbial community.

Furthermore, the lower abundance of Chloroflexi observed in the presence of *Chlorella* sp. may be linked to changes in the nature of the resources available within the digestive tract. Members of this phylum are common components of the microbiota associated with detritivorous crustaceans and participate in the degradation of complex organic matter derived from sediments and detrital material [111]. The incorporation of *Chlorella* sp. introduces an additional source of highly digestible nutrients and readily assimilable compounds, potentially modifying the nutritional profile of the intestinal contents [112]. This shift may favor bacterial groups specialized in the rapid utilization of accessible substrates, reducing the competitive advantage of taxa involved in the degradation of more recalcitrant organic compounds. Consequently, the decline of Chloroflexi may reflect a transition from a microbiota adapted to complex detrital resources toward one specialized in exploiting highly available energy sources [9].

A similar ecological mechanism may explain the lower abundance of Pirellulaceae, Halieaceae, Microbacteriaceae, Cyclobacteriaceae, and Thermoanaerobaculaceae in the presence of *Chlorella* sp. In conventional heterotrophic biofloc systems, shrimp continuously ingest aggregates rich in detritus, bacterial exopolysaccharides, and complex organic residues [113]. From an ecological perspective, the bacterial families that declined in the presence of *Chlorella* share functional traits associated with oligotrophic lifestyles and the degradation of complex organic substrates. Members of Microbacteriaceae produce chitinases and cellulases, whereas Pirellulaceae participate in the degradation of complex polysaccharides and sulfated mucins [114]. Likewise, Cyclobacteriaceae and Thermoanaerobaculaceae have been associated with the utilization of particulate organic matter and recalcitrant structural compounds present in sediments and microbial aggregates [115]. Therefore, the incorporation of *Chlorella* biomass, which provides an additional source of readily digestible nutrients, may reduce the competitive advantage of these taxa and contribute to a progressive loss of their ecological niche within the intestinal environment.

In addition, probiotic supplementation also modified the structure of the intestinal microbiota, reducing the relative abundance of Flavobacteriaceae and Thermoanaerobaculaceae. The decline of Flavobacteriaceae may be related to competitive exclusion processes, as *Bacillus* species are capable of efficiently colonizing the digestive tract and competing for both nutrients and attachment sites with other bacterial groups [116]. This finding may be particularly relevant from a health perspective, as an increase in Flavobacteriaceae within the intestinal microbiota has been proposed as a bioindicator of White Feces Syndrome (WFS) in *P. vannamei* [117]. Finally, the lower abundance of Thermoanaerobaculaceae observed in the presence of the probiotic may be associated with the colonization capacity of the *Bacillus* strains used in this study. However, given the limited knowledge currently available regarding the ecology of this bacterial family in crustaceans and the absence of studies specifically evaluating its interactions with *Bacillus*-based probiotics, this hypothesis remains speculative and warrants further investigation.

Finally, although the dominant bacterial phyla and families were largely shared between the biofloc and intestinal microbiota, their relative abundances differed considerably. This pattern suggests that the shrimp intestine is not merely a reflection of the microbial community present in the biofloc but may act as an ecological filter selecting specific bacterial groups following ingestion. While Proteobacteria increased markedly in the intestine and Planctomycetota remained relatively stable between compartments, Bacteroidota decreased substantially in the intestine compared to biofloc. In contrast, Actinobacteria and Chloroflexi became enriched in the intestinal microbiota, suggesting a greater ability of these groups to adapt to gut conditions or exploit the resources available within this environment.

A similar trend was observed at the family level. Pirellulaceae and Rhodobacteraceae maintained comparable abundances in both compartments, whereas Flavobacteriaceae decreased markedly after intestinal colonization. Conversely, Woeseiaceae and Vibrionaceae became enriched in the intestine. Together, these findings suggest that host-related factors, including nutrient availability, immune activity, and gut physicochemical conditions, play a key role in shaping the intestinal bacterial community. Therefore, although biofloc represents an important microbial reservoir, the final intestinal microbiota appears to be strongly influenced by host-mediated selection processes. Further studies are needed to better understand the mechanisms underlying this selective colonization and the functional implications of these microbial shifts for shrimp health and nutrition.

## 5. Conclusions

This study demonstrated that a single inoculation of *Chlorella* sp. was sufficient to establish the microalga within the biofloc system for approximately 40 days, although periodic reinoculations would likely be required during longer production cycles. The presence of *Chlorella* sp. improved the bioremediation capacity of the system, while both microalgae and probiotic supplementation enhanced phosphorus bioavailability in the biofloc. In addition, the microalgae increased protein bioavailability, indicating its potential to improve the nutritional quality of biofloc-derived biomass. These nutritional changes were accompanied by selective shifts in the biofloc microbiota and by the establishment of distinct intestinal bacterial communities. The lower growth performance and survival observed in the microalgae treatments may have been associated with the elevated nitrate concentrations introduced with the microalgal inoculum, highlighting the need to explore alternative inoculation strategies that minimize nutrient carry-over from the culture medium. Despite these beneficial effects, the probiotic and control treatments showed comparable zootechnical performance, and both outperformed the *Chlorella* sp. treatments. Nevertheless, probiotic supplementation increased shrimp crude protein content and reduced the abundance of *Flavobacteriaceae*, a bacterial family proposed as a bioindicator of White Feces Syndrome (WFS) in *P. vannamei*, highlighting its potential relevance for shrimp health. Overall, the results demonstrate that microorganism supplementation can be used to modulate both the nutritional and microbial characteristics of biofloc systems, with potential implications for nutrient recycling and health management.

## Figures and Tables

**Table 1:** Proximate composition and energy content of biofloc.

	Dry Matter	Crude Protein	Crude Lipid	Ash	Energy (kJ g^−1^)
M	95.8 ± 0.63	18.57 ± 0.45 ^a^	1.47 ± 0.49	49.3 ± 1.62 ^ab^	2.57 ± 0.05
MP	94.5 ± 0.69	24.42 ± 0.36 ^b^	2.90 ± 0.03	47.5 ± 2.00 ^a^	2.92 ± 0.02
P	95.7 ± 1.12	22.08 ± 0.39 ^b^	1.85 ± 0.84	50.1 ± 0.58 ^ab^	2.87 ± 0.28
C	95.4 ± 1.09	23.89 ± 0.61 ^b^	2.83 ± 0.14	51.1 ± 2.92 ^b^	2.92 ± 0.03

Energy was calculated according to [45], from the C (g) and N (g) balance (GE = 51.8 × C − 19.4 × N). Values are presented as mean ± standard error of the mean (*n* = 3). Microalgae (*Chlorella* sp.) (M), microalgae (*Chlorella* sp.) + bacterial probiotics (MP), bacterial probiotics (P), and control (C). Different letters indicate significant differences between treatments (*p* < 0.05). The means comparison test used was Student–Newman–Keuls.

**Table 2:** Main differences (RA%) between treatments and between *Chlorella* sp. and bacterial probiotics presence/absence groups of biofloc bacterial populations.

	Initial	Final			
**PHYLUM**		**M**	**MP**	**P**	**C**
Acidobacteriota	0.14 ± 0.02	10.96 ± 2.31 *	5.97 ± 1.48 *	13.02 ± 1.02 *	21.16 ± 7.36 *
		***Chlorella* sp.**		**Bacterial Probiotics**	
**PHYLUM**		**Chs**	**N-Chs**	**Pbs**	**N-Pbs**
Actinobacteria		0.78 ± 0.04 ^a^	4.49 ± 1.64 ^b^	2.04 ± 1.83	8.09 ± 3.43
**FAMILY**					
Rhodobacteraceae		9.52 ± 2.10	9.35 ± 0.09	10.15 ± 1.21 ^b^	8.72 ± 0.97 ^a^
Cyclobacteriaceae		2.30 ± 0.17 ^a^	3.23 ± 0.38 ^b^	2.96 ± 0.77	2.57 ± 0.56

Treatments with *Chlorella* (Chs: M + MP) and without *Chlorella* (N-Chs: P + C); treatments with probiotics (Pbs: MP + P) and without probiotics (N-Pbs: M + C). Microalgae (*Chlorella* sp.) (M), microalgae (*Chlorella* sp.) + bacterial probiotics (MP), bacterial probiotics (P), and control (C). Values represented as mean ± standard error of the mean (*n* = 3). Different letters indicate significant differences between final treatments. “*” indicates significant differences with initial values for each treatment (*p* < 0.05).

**Table 3:** Zootechnical parameters of shrimps.

	M	MP	P	C
**Initial weight (g)**	2.4 ± 0.0	2.5 ± 0.0	2.5 ± 0.0	2.6 ± 0.1
**Final weight (g)**	7.1 ± 0.0 ^a^	7.9 ± 0.5 ^a^	9.6 ± 0.2 ^b^	9.0 ± 0.5 ^b^
**Survival rate (%)**	45.8 ± 8.7 ^a^	57.2 ± 26.6 ^a^	77.9 ± 6.6 ^b^	91.2 ± 8.7 ^b^
**SGR (% day^−1^)**	1.8 ± 0.0 ^a^	1.9 ± 0.1 ^ab^	2.2 ± 0.0 ^c^	2.0 ± 0.1 ^bc^
**FCR** (g feed g^−1^ gain)	2.2 ± 0.3 ^c^	1.9 ± 0.3 ^bc^	1.3 ± 0.2 ^a^	1.6 ± 0.2 ^ab^
**PER**	1.2 ± 0.1 ^a^	1.4 ± 0.2 ^a^	2.0 ± 0.3 ^b^	1.7 ± 0.2 ^ab^

Values are presented as mean ± standard error of the mean (*n* = 3). For each treatment, the values that do not share the same letter are significantly different (*p* < 0.05). Microalgae (*Chlorella* sp.) (M), microalgae (*Chlorella* sp.) + bacterial probiotics (MP), bacterial probiotics (P), and control (C).Survival rate = (final number animals/initial number animals) × 100) (%) (S); (specific growth rate (SGR) = 100 × ln (final weight/initial weight)/days; feed conversion ratio (FCR) = feed consumption (g)/biomass gain (g); protein efficiency ratio (PER) = gain weight (g)/consumed protein (g).

**Table 4:** Proximate composition and energy content of whole-body (wet weight basis) of *P. vannamei*.

	Dry Matter %	Crude Protein (%)	Crude Lipid (%)	Ash (%)	Energy (kJ g^−1^) *
**M**	78.83 ± 0.81	18.62 ± 0.80 ^a^	1.38 ± 0.20	3.03 ± 0.07	3.16 ± 0.07
**MP**	78.42 ± 0.88	18.22 ± 0.65 ^a^	1.34 ± 0.55	3.05 ± 0.10	3.05 ± 0.10
**P**	75.84 ± 1.35	20.44 ± 1.49 ^b^	1.56 ± 0.92	3.14 ± 0.44	3.17 ± 0.07
**C**	78.20 ± 0.39	18.96 ± 0.35 ^a^	1.73 ± 0.38	2.97 ± 0.16	3.11 ± 0.16

* Energy was calculated according to [45], from the C (g) and N (g) balance (GE = 51.8 × C − 19.4 × N). Microalgae (*Chlorella* sp.) (M), microalgae (*Chlorella* sp.) + bacterial probiotics (MP), bacterial probiotics (P), and control (C). Values are presented as mean ± standard error of the mean (*n* = 3). Different letters indicate significant differences between treatments (*p* < 0.05).

**Table 5:** Main differences (RA%) between treatments and between *Chlorella* sp. and bacterial probiotics presence/absence groups of shrimp gut bacterial populations.

	Initial	Final			
**PHYLUM**		**M**	**MP**	**P**	**C**
Actinobacteria	3.27 ± 0.68	8.45 ± 2.48 *	7.09 ± 2.76 *	7.74 ± 2.50 *	9.97 ± 2.84 *
Chloroflexi	2.26 ± 0.43	5.11 ± 1.62 *	5.97 ± 2.56 *	6.44 ± 2.69 *	8.67 ± 3.70 *
Acidobacteriota	1.27 ± 0.14	0.25 ± 0.18 ^a^*	0.45 ± 0.40 ^a^*	0.51 ± 0.23 ^a^*	3.78 ± 2.52 ^b^*
**FAMILY**					
Pirellulaceae	20.44 ± 3.69	12.50 ± 4.42 ^a^	12.96 ± 5.66 ^ab^	15.26 ± 6.11 ^ab^	18.14 ± 3.36 ^b^
Halieaceae	7.62 ± 1.86	1.86 ± 0.70 ^a^*	2.35 ±0.50 ^ab^*	2.86 ± 0.91 ^ab^*	3.87 ± 1.47 ^b^*
Microbacteriaceae	0.16 ± 0.06	2.23 ± 0.64 ^a^*	1.68 ± 0.79 ^a^*	3.92 ± 0.77 ^b^*	3.85 ± 0.34 ^b^*
Cyclobacteriaceae	2.28 ± 0.32	0.92 ± 0.36 ^a^*	2.57 ±0.49 ^ab^	1.37 ± 0.69 ^ab^	2.85 ± 0.46 ^b^
Thermoanaerobaculaceae	0.86 ± 0.14	0.07 ± 0.04 ^a^*	0.20 ± 0.17 ^b^*	0.51 ± 0.23 ^b^*	4.73 ± 2.70 ^c^*
		***Chlorella* sp.**		**Bacterial Probiotics**	
**PHYLUM**		**Chs**	**N-Chs**	**Pbs**	**N-Pbs**
Acidobacteriota		0.28 ± 0.03 ^a^	1.07 ± 0.26 ^b^	0.35 ± 0.04 ^a^	1.97 ± 0.59 ^b^
Chloroflexi		5.54 ± 2.18 ^a^	7.55 ± 3.24 ^b^	6.21 ± 2.68	6.89 ± 3.37
**FAMILY**					
Microbacteriaceae		1.95 ± 0.39 ^a^	3.88 ± 0.05 ^b^	2.80 ± 1.58	3.04 ± 1.15
Thermoanaerobaculaceae		0.28 ± 0.06 ^a^	2.40 ± 1.07 ^b^	0.39 ± 0.01 ^a^	2.55 ± 1.97 ^b^
Flavobacteriaceae		5.27 ± 1.55	3.65 ± 0.49	3,74 ± 0.61 ^a^	5.18 ± 1.68 ^b^

Treatments with Chlorella (Chs: M + MP) and without Chlorella (N-Chs: P + C); treatments with probiotics (Pbs: MP + P) and without probiotics (N-Pbs: M + C) Microalgae (*Chlorella* sp.) (M), microalgae (*Chlorella* sp.) + bacterial probiotics (MP), bacterial probiotics (P), and control (C). Values represented as mean ± standard error of the mean (*n* = 3). Different letters indicate significant differences between final treatments. “*” indicates significant differences with initial values for each treatment (*p* < 0.05).

## Data Availability

The data presented in this study are available in the RiuNet Institutional Repository of the Universitat Politècnica de València: https://riunet.upv.es/handle/10251/238977 (accessed on 3 August 2026).

## Supplementary Materials

The following supporting information can be downloaded at: https://www.mdpi.com/article/10.3390/ani16162469/s1, Table S1: Proximate composition of the commercial feed. Table S2: Multifactorial parametric ANOVA results for chlorophyll concentration considering treatment and sampling time. Chlorophyll values represent the mean concentration throughout the experimental period for each treatment. Table S3: Water quality of treatments. Table S4: Summary of the quality of 16S rRNA gene sequencing of biofloc and shrimp intestine samples at the beginning and end of the experiment. Table S5: Alpha diversity indexes of phylum and family taxonomic level from biofloc microbiota. Table S6: Alpha diversity indexes of family taxonomic level from shrimp intestine microbiota. Table S7: PERMANOVA results based on Bray–Curtis dissimilarities showing the effects of treatment on bacterial beta diversity in biofloc and shrimp intestine samples at the phylum and family taxonomic levels.

## Abbreviations

The following abbreviations are used in this manuscript:

AOAC	Association of Official Analytical Chemists
ASVs	Amplicon sequence variants
BFT	Biofloc technology
CFU	Colony-forming unit
CLR	Centered log-ratio
DO	Dissolved oxygen
PCR	Polymerase chain reaction
PCoA	Principal coordinates analysis
PERMANOVA	Permutational multivariate analysis of variance
PSU	Practical salinity unit
RA	Relative abundance
TAN	Total ammonia nitrogen
TSS	Total suspended solids
WFS	White Feces Syndrome

## References

[1] Khanjani M.H., Sharifinia M. (2020). Biofloc Technology as a Promising Tool to Improve Aquaculture Production. Rev. Aquac..

[2] Avnimelech Y. (2012). Biofloc Technology—A Practical Guide Book.

[3] Angela D., Arbi S., Natrah F.M.I., Widanarni W., Pande G.S.J., Ekasari J. (2021). Evaluation of *Chlorella* sp. and *Ankistrodesmus* sp. Addition on Biofloc System Performance in Giant Prawn Culture. Aquac. Res..

[4] Ray A.J., Seaborn G., Vinatea L., Browdy C.L., Leffler J.W. (2012). Effects of Biofloc Reduction on Microbial Dynamics in Minimal-Exchange, Superintensive Shrimp, *Litopenaeus vannamei*, Culture Systems. J. World Aquac. Soc..

[5] Said M.M., Abo-Al-Ela H.G., El-Barbary Y.A., Ahmed O.M., Dighiesh H.S. (2024). Influence of Stocking Density on the Growth, Immune and Physiological Responses, and Cultivation Environment of White-Leg Shrimp (*Litopenaeus vannamei*) in Biofloc Systems. Sci. Rep..

[6] Liu G., Zhu S., Liu D., Guo X., Ye Z. (2017). Effects of Stocking Density of the White Shrimp *Litopenaeus vannamei* (Boone) on Immunities, Antioxidant Status, and Resistance against *Vibrio harveyi* in a Biofloc System. Fish. Shellfish Immunol..

[7] Butt U.D., Lin N., Akhter N., Siddiqui T., Li S., Wu B. (2021). Overview of the Latest Developments in the Role of Probiotics, Prebiotics and Symbiotic in Shrimp Aquaculture. Fish. Shellfish Immunol..

[8] Kathia C.M., del Carmen M.D.M., Aida H.P., Jorge C.M., Daniel B.C. (2017). Probiotics Used in Biofloc System for Fish and Crustacean Culture: A Review. Aquaculture.

[9] Raza B., Zheng Z., Zhu J., Yang W. (2024). A Review: Microbes and Their Effect on Growth Performance of *Litopenaeus vannamei* (White Leg Shrimps) during Culture in Biofloc Technology System. Microorganisms.

[10] Martínez-Córdova L.R., Emerenciano M., Miranda-Baeza A., Martínez-Porchas M. (2015). Microbial-Based Systems for Aquaculture of Fish and Shrimp: An Updated Review. Rev. Aquac..

[11] Ferreira M.G.P., Melo F.P., Lima J.P.V., Andrade H.A., Severi W., Correia E.S. (2017). Bioremediation and Biocontrol of Commercial Probiotic in Marine Shrimp Culture with Biofloc. Lat. Am. J. Aquat. Res..

[12] Hostins B., Lara G., Decamp O., Cesar D.E., Wasielesky W. (2017). Efficacy and Variations in Bacterial Density in the Gut of *Litopenaeus vannamei* Reared in a BFT System and in Clear Water Supplemented with a Commercial Probiotic Mixture. Aquaculture.

[13] Kongnum K., Hongpattarakere T. (2012). Effect of *Lactobacillus plantarum* Isolated from Digestive Tract of Wild Shrimp on Growth and Survival of White Shrimp (*Litopenaeus vannamei*) Challenged with *Vibrio harveyi*. Fish. Shellfish Immunol..

[14] Madani N.S.H., Adorian T.J., Farsani H.G., Hoseinifar S.H. (2018). The Effects of Dietary Probiotic Bacilli (*Bacillus subtilis* and *Bacillus licheniformis*) on Growth Performance, Feed Efficiency, Body Composition and Immune Parameters of Whiteleg Shrimp (*Litopenaeus vannamei*) Postlarvae. Aquac. Res..

[15] Ferreira G.S., Bolívar N.C., Pereira S.A., Guertler C., do Nascimento Vieira F., Mouriño J.L.P., Seiffert W.Q. (2015). Microbial Biofloc as Source of Probiotic Bacteria for the Culture of *Litopenaeus vannamei*. Aquaculture.

[16] Menaga M., Rajasulochana P., Felix S., Sudarshan S., Kapoor A., Gandla K., Saleh M.M., Ibrahim A.E., El Deeb S. (2023). Evaluation of Biofloc-Based Probiotic Isolates on Growth Performance and Physiological Responses in *Litopenaeus vannamei*. Water.

[17] Ma M., Hu Q. (2024). Microalgae as Feed Sources and Feed Additives for Sustainable Aquaculture: Prospects and Challenges. Rev. Aquac..

[18] Meenakshisundaram M., Mboya J.B., Sugantham F., Panigrahi A., Gamba J.L., Subramanian S., Chia S.Y., Beesigamukama D., Munguti J., Ogello E. (2025). Synergistic Microbial Interactions Between Algae and Bacteria Augment Growth and Immune Performance in Red Tilapia (*Oreochromis* sp.). Aquac. J..

[19] Yu Y.B., Choi J.H., Lee J.H., Jo A.H., Lee J.W., Choi H.J., Kang Y.J., Choi C.Y., Kang J.C., Lee K.M. (2024). The Use, Application and Efficacy of Biofloc Technology (BFT) in Shrimp Aquaculture Industry: A Review. Environ. Technol. Innov..

[20] Natrah F.M.I., Bossier P., Sorgeloos P., Yusoff F.M., Defoirdt T. (2014). Significance of Microalgal-Bacterial Interactions for Aquaculture. Rev. Aquac..

[21] Charoonnart P., Purton S., Saksmerprome V. (2018). Applications of Microalgal Biotechnology for Disease Control in Aquaculture. Biology.

[22] de Moraes L.B.S., Galvez A.O. (2020). Advances in the Use of Photoheterotrophic, Mixotrophic and Multi-Trophic Systems in Marine Shrimp Farming. Borneo J. Mar. Sci. Aquac. (BJoMSA).

[23] Liu J., Chen F. (2014). Biology and Industrial Applications of *Chlorella*: Advances and Prospects. Microalgae Biotechnol..

[24] Safi C., Zebib B., Merah O., Pontalier P.-Y., Vaca-Garcia C. (2014). Morphology, Composition, Production, Processing and Applications of *Chlorella vulgaris*: A Review. Renew. Sustain. Energy Rev..

[25] Cascales-Martos T., Aguilar-Escribano J., Brol J., Jover-Cerdá M., Martínez-Llorens S., Tomás-Vidal A., Sánchez-Jerez P., Peñaranda D.S. (2026). Biofloc Characterization after Achieving a Stable Population of *Chlorella sp*. for *Penaeus vannamei* Culture (Bonne, 1931). Aquac. Rep..

[26] Jung J.Y., Damusaru J.H., Park Y., Kim K., Seong M., Je H.W., Kim S., Bai S.C. (2017). Autotrophic Biofloc Technology System (ABFT) Using *Chlorella vulgaris* and *Scenedesmus obliquus* Positively Affects Performance of Nile Tilapia (*Oreochromis niloticus*). Algal Res..

[27] Dong S., Li Y., Huang F., Lin L., Li Z., Li J., Zhang Y., Zheng Y. (2022). Enhancing Effect of *Platymonas* Addition on Water Quality, Microbial Community Diversity and Shrimp Performance in Biofloc-Based Tanks for *Penaeus vannamei* Nursery. Aquaculture.

[28] Eissa E.S.H., Aljarari R.M., Elfeky A., Abd El-Aziz Y.M., Munir M.B., Jastaniah S.D., Alaidaroos B.A., Shafi M.E., Abd El-Hamed N.N.B., AL-Farga A. (2023). Protective Effects of *Chlorella vulgaris* as a Feed Additive on Growth Performance, Immunity, Histopathology, and Disease Resistance against *Vibrio parahaemolyticus* in the Pacific White Shrimp. Aquac. Int..

[29] Qingman C., Chunying Y., Wenhao Z., Wenxue L. (2019). Effects of *Chlorella vulgaris* on Immuno-Related Factors and Their Expression in *Penaeus vannamei*. Isr. J. Aquac.-Bamidgeh.

[30] Khanjani M.H., Mohammadi A., Emerenciano M.G.C. (2024). Water Quality in Biofloc Technology (BFT): An Applied Review for an Evolving Aquaculture. Aquac. Int..

[31] Fu L.L., Chen X., Wang Y. (2014). Quality Evaluation of Farmed Whiteleg Shrimp, *Litopenaeus vannamei*, Treated with Different Slaughter Processing by Infrared Spectroscopy. Food Chem..

[32] Frozza A., Fiorini A., Vendruscolo E.C.G., Rosado F.R., Konrad D., Rodrigues M.C.G., Ballester E.L.C. (2021). Probiotics in the Rearing of Freshwater Prawn *Macrobrachium rosenbergii* (de Man, 1879) in a Biofloc System. Aquac. Res..

[33] Jory D., Cabrera T., Dugger D., Fegan D., Berger C., Orrantia J., Wainberg A., Perez H., Castañeda J., McIntosh R. (2001). A Global Review of Shrimp Feed Management: Status and Perspectives. Aquac. 2001 Book Abstr..

[34] Strickland J.D.H., Parsons T.R., Stevenson J.C., Watson J., Reinhart J.M., CooK D.G. (1972). A Practical Handbook of Seawater Analysis.

[35] Koroleff F., Grasshoff K. (1976). Methods of Seawater Analysis.

[36] Miranda K.M., Espey M.G., Wink D.A. (2001). A Rapid, Simple Spectrophotometric Method for Simultaneous Detection of Nitrate and Nitrite. Nitric Oxide.

[37] Ebeling J.M., Timmons M.B., Bisogni J.J. (2006). Engineering Analysis of the Stoichiometry of Photoautotrophic, Autotrophic, and Heterotrophic Removal of Ammonia–Nitrogen in Aquaculture Systems. Aquaculture.

[38] Hand E., Stein J.R. (1973). Pigment Analysis. Handbook of Phycological Methods, Culture Methods and Growth Measurements.

[39] Engelen A.H., Peene J., Lancelot C. (1994). A Rapid Method for the Determination of Total Phosphorus in Marine Particulate Material by Acid Digestion and Spectrophotometric Analysis. Hydrobiologia.

[40] Kureshy N., Allen Davis D. (2002). Protein Requirement for Maintenance and Maximum Weight Gain for the Pacific White Shrimp, *Litopenaeus vannamei*. Aquaculture.

[41] National Research Council (2011). Nutrient Requirements of Fish and Shrimp.

[42] De Silva S.S., Anderson T.A. (1995). Fish Nutrition in Aquaculture.

[43] Chapman D.G., Castillo R., Campbell J.A. (1959). Evaluation of Protein in Foods: A Method for the Determination of Protein Efficiency Ratios. Can. J. Biochem. Physiol..

[44] Church F.C., Swaisgood H.E., Porter D.H., Catignani G.L. (1983). Spectrophotometric Assay Using O-Phthaldialdehyde for Determination of Proteolysis in Milk and Isolated Milk Proteins. J. Dairy Sci..

[45] Brouwer E. (1965). Report of Sub-Committee on Constants and Factors. Proceedings of the 3rd Symposium on Energy Metabolism.

[46] Aly S.M., ElBanna N.I., Fathi M. (2023). Chlorella in Aquaculture: Challenges, Opportunities, and Disease Prevention for Sustainable Development. Aquac. Int..

[47] Ekasari J., Nugroho U.A., Fatimah N., Angela D., Hastuti Y.P., Pande G.S.J., Natrah F.M.I. (2021). Improvement of Biofloc Quality and Growth of *Macrobrachium rosenbergii* in Biofloc Systems by *Chlorella* Addition. Aquac. Int..

[48] Xiao L., Chen Z., Yang Y., Liu Z. (2022). Growth Promotion of *Chlorella* by Symbiotic Bacteria under Adverse Environments. Algal Res..

[49] Guo Z., Tong Y.W. (2014). The Interactions between *Chlorella vulgaris* and Algal Symbiotic Bacteria under Photoautotrophic and Photoheterotrophic Conditions. J. Appl. Phycol..

[50] Liang Z., Liu Y., Ge F., Xu Y., Tao N., Peng F., Wong M. (2013). Efficiency Assessment and PH Effect in Removing Nitrogen and Phosphorus by Algae-Bacteria Combined System of *Chlorella vulgaris* and *Bacillus licheniformis*. Chemosphere.

[51] Castro-Mejía G., de Lara A.R., Monroy-Dosta M.C., Maya-Gutiérrez S., Castro-Mejía J., Jiménez-Pacheco F. (2017). Presencia y Abundancia de Fitoplancton y Zooplancton en un Sistema de Producción de Biofloc Utilizando dos Aportes de Carbono: 1) Melaza y 2) Melaza + Pulido de Arroz Cultivando al Pez Oreochromis niloticus. Rev. Digit. Dep. Hombre Su Ambiente.

[52] Ge H., Li J., Chang Z., Chen P., Shen M., Zhao F. (2016). Effect of Microalgae with Semicontinuous Harvesting on Water Quality and Zootechnical Performance of White Shrimp Reared in the Zero Water Exchange System. Aquac. Eng..

[53] Huang C., Luo Y., Zeng G., Zhang P., Peng R., Jiang X., Jiang M. (2022). Effect of Adding Microalgae to Whiteleg Shrimp Culture on Water Quality, Shrimp Development and Yield. Aquac. Rep..

[54] Luo G., Xu J., Meng H. (2020). Nitrate Accumulation in Biofloc Aquaculture Systems. Aquaculture.

[55] Marinho Y.F., Brito L.O., da Silva Campos C.V.F., Severi W., Andrade H.A., Galvez A.O. (2017). Effect of the Addition of *Chaetoceros calcitrans*, *Navicula* sp. and *Phaeodactylum tricornutum* (Diatoms) on Phytoplankton Composition and Growth of *Litopenaeus vannamei* (Boone) Postlarvae Reared in a Biofloc System. Aquac. Res..

[56] Ding Y., Chen N., Ke J., Qi Z., Chen W., Sun S., Zheng Z., Xu J., Yang W. (2021). Response of the Rearing Water Bacterial Community to the Beneficial Microalga *Nannochloropsis oculata* Cocultured with Pacific White Shrimp (*Litopenaeus vannamei*). Aquaculture.

[57] Miranda-Baeza A., Nolasco-López M., Rivas-Vega M.E., Huerta-Rábago J.A., Martínez-Córdova L.R., Martínez-Porchas M. (2020). Short-Term Effect of the Inoculation of Probiotics in Mature Bioflocs: Water Quality Parameters and Abundance of Heterotrophic and Ammonia-Oxidizing Bacteria. Aquac. Res..

[58] Neto H.S., Santaella S.T., Nunes A.J.P. (2015). Bioavailability of Crude Protein and Lipid from Biofloc Meals Produced in an Activated Sludge System for White Shrimp, *Litopenaeus vannamei*. Rev. Bras. Zootec..

[59] Kumar V., Roy S., Behera B.K., Swain H.S., Das B.K. (2021). Biofloc Microbiome with Bioremediation and Health Benefits. Front. Microbiol..

[60] Kandathil Radhakrishnan D., AkbarAli I., Schmidt B.V., John E.M., Sivanpillai S., Thazhakot Vasunambesan S. (2020). Improvement of Nutritional Quality of Live Feed for Aquaculture: An Overview. Aquac. Res..

[61] Ajomiwe N., Boland M., Phongthai S., Bagiyal M., Singh J., Kaur L. (2024). Protein Nutrition: Understanding Structure, Digestibility, and Bioavailability for Optimal Health. Foods.

[62] Venkata Subhash G., Chugh N., Iyer S., Waghmare A., Musale A.S., Nandru R., Dixit R.B., Gaikwad M.S., Menon D., Thorat R. (2020). Application of in Vitro Protein Solubility for Selection of Microalgae Biomass as Protein Ingredient in Animal and Aquafeed. J. Appl. Phycol..

[63] Faramudhita I., Widanarni, Yuhana M., Gustilatov M., Rahmawati R. (2026). Comparative Evaluation of Biofloc-Derived Probiotics on Growth Performance, Immune Response, and Intestinal Microbiota Modulation in *Penaeus vannamei*. Aquac. Rep..

[64] Ahmad A., Osman S.M., Cha T.S., Loh S.H. (2017). Phosphate-Induced Changes in Fatty Acid Biosynthesis in *Chlorella* sp. KS-MA2 Strain. BioTechnologia.

[65] Ahmad M.T., Shariff M., Yusoff F.M., Goh Y.M., Banerjee S. (2020). Applications of Microalga *Chlorella vulgaris* in Aquaculture. Rev. Aquac..

[66] Choi Y.S., Hong S.W., Kim S.J., Chung I.H. (2002). Development of a Biological Process for Livestock Wastewater Treatment Using a Technique for Predominant Outgrowth of *Bacillus* Species. Water Sci. Technol..

[67] Hlordzi V., Kuebutornye F.K.A., Afriyie G., Abarike E.D., Lu Y., Chi S., Anokyewaa M.A. (2020). The Use of *Bacillus* Species in Maintenance of Water Quality in Aquaculture: A Review. Aquac. Rep..

[68] Cheng K.M., Hu C.Q., Liu Y.N., Zheng S.X., Qi X.J. (2006). Effects of Dietary Calcium, Phosphorus and Calcium/Phosphorus Ratio on the Growth and Tissue Mineralization of *Litopenaeus vannamei* Reared in Low-Salinity Water. Aquaculture.

[69] Coelho R., Reynaud J.G., Biard C., Ribeiro B., Lemos D. (2024). Inorganic Phosphorus Supplementation in Diets for Farmed Shrimp: Performance and Digestibility of Juvenile *Litopenaeus vannamei* Fed Monosodium Phosphate, Monoammonium Phosphate, Magnesium Phosphate, and Monocalcium Phosphate. Aquac. Res..

[70] Rajeev M., Jung I., Kang I., Cho J.-C., Poretsky R. (2024). Genome-Centric Metagenomics Provides Insights into the Core Microbial Community and Functional Profiles of Biofloc Aquaculture. mSystems.

[71] Baskaran V., Patil P.K., Leo Antony M., Avunje S., Nagaraju V.T., Ghate S.D., Nathamuni S., Dineshkumar N., Alavandi S.V., Vijayan K.K. (2020). Microbial Community Profiling of Ammonia and Nitrite Oxidizing Bacterial Enrichments from Brackishwater Ecosystems for Mitigating Nitrogen Species. Sci. Rep..

[72] Zhao Z., Zhao Y., Marotta F., Xamxidin M., Li H., Xu J., Hu B., Wu M. (2024). The Microbial Community Structure and Nitrogen Cycle of High-Altitude Pristine Saline Lakes on the Qinghai-Tibetan Plateau. Front. Microbiol..

[73] Wang Y., Shi X., Zhao S., Sun B., Liu Y., Li W., Yu H., Tian Z., Guo X., Shi Y. (2023). Characterizing Free-Living and Particle-Attached Bacterial Communities of a Shallow Lake on the Inner Mongolia-Xinjiang Plateau, China. Water.

[74] Lian C., Xiang J., Cai H., Ke J., Ni H., Zhu J., Zheng Z., Lu K., Yang W. (2024). Microalgae Inoculation Significantly Shapes the Structure, Alters the Assembly Process, and Enhances the Stability of Bacterial Communities in Shrimp-Rearing Water. Biology.

[75] Fitzgerald C.M., Camejo P., Oshlag J.Z., Noguera D.R. (2014). Ammonia-Oxidizing Microbial Communities in Reactors with Efficient Nitrification at Low-Dissolved Oxygen. Water Res..

[76] Gong Y., Ping X.Y., Zeng C.H., Wang S.X., Zhou Y., Wang M.Y., Mu D.S., Du Z.J. (2022). Predation Capacity of Bradymonabacteria, a Recently Discovered Group in the Order Bradymonadales, Isolated from Marine Sediments. Arch. Microbiol..

[77] Vinasyiam A. (2025). Influence of Carbon Source Complexity on Prokaryotic Community Composition in Biofloc System: A Review. J. Perikan. Unram.

[78] Chan-Vivas E., Edén M.G., Maldonado C., Escalante K., Gaxiola G., Cuzon G. (2019). Does Biofloc Improve the Energy Distribution and Final Muscle Quality of Shrimp, *Litopenaeus vannamei* (Boone, 1883)?. J. World Aquac. Soc..

[79] Khanjani M.H., Alizadeh M., Sharifinia M. (2020). Rearing of the Pacific White Shrimp, *Litopenaeus vannamei* in a Biofloc System: The Effects of Different Food Sources and Salinity Levels. Aquac. Nutr..

[80] Luis-Villaseñor I.E., Voltolina D., Audelo-Naranjo J.M., Pacheco-Marges M.R., Herrera-Espericueta V.E., Romero-Beltrán E. (2015). Effects of Biofloc Promotion on Water Quality, Growth, Biomass Yield and Heterotrophic Community in *Litopenaeus vannamei* (Boone, 1931) Experimental Intensive Culture. Ital. J. Anim. Sci..

[81] Zhao D., Pan L., Huang F., Wang C., Xu W. (2016). Effects of Different Carbon Sources on Bioactive Compound Production of Biofloc, Immune Response, Antioxidant Level, and Growth Performance of *Litopenaeus vannamei* in Zero-Water Exchange Culture Tanks. J. World Aquac. Soc..

[82] Ramasubburayan R., Prakash S., Immanuel G., Mubarakali D., Rajakumar G., Thirumurugan D., Palavesam A. (2025). The Transformative Role of Prebiotics, Probiotics, and Microbiomes in Biofloc Systems for Sustainable Aquaculture: A Comprehensive Review. Rev. Aquac..

[83] Daniel N., Nageswari P. (2017). Exogenous Probiotics on Biofloc Based Aquaculture: A Review. Curr. Agric. Res. J..

[84] Krummenauer D., Poersch L., Romano L.A., Lara G.R., Encarnação P., Wasielesky W. (2014). The Effect of Probiotics in a *Litopenaeus vannamei* Biofloc Culture System Infected with *Vibrio parahaemolyticus*. J. Appl. Aquac..

[85] Nitsos C., Filali R., Taidi B., Lemaire J. (2020). Current and Novel Approaches to Downstream Processing of Microalgae: A Review. Biotechnol. Adv..

[86] Lavens P., Sorgeloos P. (1996). Manual on the Production and Use of Live Food for Aquaculture.

[87] Najjar Y.S.H., Abu-Shamleh A. (2020). Harvesting of Microalgae by Centrifugation for Biodiesel Production: A Review. Algal Res..

[88] Prates E., Okamoto M., Holanda M., Monserrat J.M., Slater M.J., Wasielesky W. (2024). Determination of Acute Toxicity and Evaluation of the Chronic Effect of Nitrate on Compensatory Growth of *Litopenaeus vannamei* Reared in a Biofloc Technology System. Aquaculture.

[89] Valencia-Castañeda G., Frías-Espericueta M.G., Vanegas-Pérez R.C., Chávez-Sánchez M.C., Páez-Osuna F. (2019). Toxicity of Ammonia, Nitrite and Nitrate to *Litopenaeus vannamei* Juveniles in Low-Salinity Water in Single and Ternary Exposure Experiments and Their Environmental Implications. Environ. Toxicol. Pharmacol..

[90] Neto I.A., Brandão H., Furtado P.S., Wasielesky W. (2019). Acute Toxicity of Nitrate in *Litopenaeus vannamei* Juveniles at Low Salinity Levels. Ciência Rural.

[91] Dutra F.M., Rio G.S., Zadinelo I.V., Cupertino Ballester E.L. (2020). Exposure of *Macrobrachium rosenbergii* (De Man, 1879) Post-Larvae to Different Nitrate Concentrations: Effect on Performance and Welfare. Aquaculture.

[92] Singh G., Patidar S.K. (2018). Microalgae Harvesting Techniques: A Review. J. Environ. Manag..

[93] Abdelaziz A.E.M., Leite G.B., Hallenbeck P.C. (2013). Addressing the Challenges for Sustainable Production of Algal Biofuels: II. Harvesting and Conversion to Biofuels. Environ. Technol..

[94] Barros A.I., Gonçalves A.L., Simões M., Pires J.C.M. (2015). Harvesting Techniques Applied to Microalgae: A Review. Renew. Sustain. Energy Rev..

[95] Chen Y., Fu Z., Shen Z., Zhang R., Zhao J., Zhang Y., Xu Q. (2023). Rapid Production Biofloc by Inoculating *Chlorella pyrenoidosa* in a Separate Way. Water.

[96] Aliasgari E. (2024). Investigation of Probiotic’s Effect on Efficacy of Biofloc in Shrimp Farming. Agric. Mark. Commer..

[97] Huang Z., Li X., Wang L., Shao Z. (2016). Changes in the Intestinal Bacterial Community during the Growth of White Shrimp, *Litopenaeus vannamei*. Aquac. Res..

[98] Li E., Xu C., Wang X., Wang S., Zhao Q., Zhang M., Qin J.G., Chen L. (2018). Gut Microbiota and Its Modulation for Healthy Farming of Pacific White Shrimp *Litopenaeus vannamei*. Rev. Fish. Sci. Aquac..

[99] Qiao L., Bao J.J., Li T.J., Sun X.M., Ze W.G., Li J. (2025). Microbial Community Structure and Its Driving Factors in the Whiteleg Shrimp (*Litopenaeus vannamei*) Aquaculture System. Appl. Ecol. Environ. Res..

[100] Rajeev M., Jung I., Song J., Kang I., Cho J.C. (2023). Comparative Microbiota Characterization Unveiled a Contrasting Pattern of Floc-Associated versus Free-Living Bacterial Communities in Biofloc Aquaculture. Aquaculture.

[101] Wang R., Chen H., Zhu Y., Al-Masqari Z.A., Yan M., Wang G., Dong P., Gao F., Lu T., Zhang D. (2022). Survival Status of *Penaeus vannamei* Is Associated with the Homeostasis and Assembly Process of the Intestinal Bacterial Community. Aquaculture.

[102] Zeng S., Huang Z., Hou D., Liu J., Weng S., He J. (2017). Composition, Diversity and Function of Intestinal Microbiota in Pacific White Shrimp (*Litopenaeus vannamei*) at Different Culture Stages. PeerJ.

[103] Landsman A., St-Pierre B., Rosales-Leija M., Brown M., Gibbons W. (2019). Impact of Aquaculture Practices on Intestinal Bacterial Profiles of Pacific Whiteleg Shrimp *Litopenaeus vannamei*. Microorganisms.

[104] Klimek D., Herold M., Calusinska M. (2024). Comparative Genomic Analysis of Planctomycetota Potential for Polysaccharide Degradation Identifies Biotechnologically Relevant Microbes. BMC Genom..

[105] Fan L., Li Q.X. (2019). Characteristics of Intestinal Microbiota in the Pacific White Shrimp *Litopenaeus vannamei* Differing Growth Performances in the Marine Cultured Environment. Aquaculture.

[106] Li H., Gu S., Wang L., Shi W., Jiang Q., Wan X. (2024). Dynamic Changes of Environment and Gut Microbial Community of *Litopenaeus vannamei* in Greenhouse Farming and Potential Mechanism of Gut Microbial Community Construction. Fishes.

[107] Qiao F., Liu Y.K., Sun Y.H., Wang X.D., Chen K., Li T.Y., Li E.C., Zhang M.L. (2017). Influence of Different Dietary Carbohydrate Sources on the Growth and Intestinal Microbiota of *Litopenaeus vannamei* at Low Salinity. Aquac. Nutr..

[108] Huerta-Rábago J.A., Martínez-Porchas M., Miranda-Baeza A., Nieves-Soto M., Rivas-Vega M.E., Martínez-Córdova L.R. (2019). Addition of Commercial Probiotic in a Biofloc Shrimp Farm of *Litopenaeus vannamei* during the Nursery Phase: Effect on Bacterial Diversity Using Massive Sequencing 16S RRNA. Aquaculture.

[109] Yurgel S.N., Nadeem M., Cheema M. (2022). Microbial Consortium Associated with Crustacean Shells Composting. Microorganisms.

[110] Tong Y., Zhang M., Li Q., Hu X., Wei Y., Zhao W., Yu X., Peng M., Yang C., Chen X. (2025). Effectiveness Evaluation of Five Probiotics on Shrimp and Water Environment. Aquac. Rep..

[111] Hou D., Huang Z., Zeng S., Liu J., Weng S., He J. (2018). Comparative Analysis of the Bacterial Community Compositions of the Shrimp Intestine, Surrounding Water and Sediment. J. Appl. Microbiol..

[112] Yuan H., Song W., Tan J., Zheng Y., Wang H., Shi L., Zhang S. (2023). The Effects of Dietary Protein Level on the Growth Performance, Body Composition, Intestinal Digestion and Microbiota of *Litopenaeus vannamei* Fed *Chlorella sorokiniana* as the Main Protein Source. Animals.

[113] El-Sayed A.F.M. (2021). Use of Biofloc Technology in Shrimp Aquaculture: A Comprehensive Review, with Emphasis on the Last Decade. Rev. Aquac..

[114] Chaudhary D.K., Kim S.E., Park H.J., Kim K.H. (2024). Unveiling the Bacterial Community across the Stomach, Hepatopancreas, Anterior Intestine, and Posterior Intestine of Pacific Whiteleg Shrimp. J. Microbiol. Biotechnol..

[115] Foysal M.J. (2023). Host Habitat Shapes the Core Gut Bacteria of Decapod Crustaceans: A Meta-Analysis. Heliyon.

[116] Huang L., Guo H., Liu Z., Chen C., Wang K., Huang X., Chen W., Zhu Y., Yan M., Zhang D. (2022). Contrasting Patterns of Bacterial Communities in the Rearing Water and Gut of *Penaeus vannamei* in Response to Exogenous Glucose Addition. Mar. Life Sci. Technol..

[117] Zeng S., He J., Huang Z. (2024). The Intestine Microbiota of Shrimp and Its Impact on Cultivation. Appl. Microbiol. Biotechnol..

