# Supplementary material for: Combined and Individual Effects of Chlorella sp. and Bacterial Probiotics in Biofloc Systems for White Shrimp (Penaeus vannamei)
Source: Animals (Basel). 2026 Aug 8;16(16):2469. doi: 10.3390/ani16162469 (PMC13508912; doi:10.3390/ani16162469)
Supplement: Supplementary file 1 [file animals-16-02469-s001.zip › animals-4448346-supplementary.pdf]

Table S1: Proximal composition of commercial feed.

**Crude protein (%)**	36
**Crude lipid (%)**	10
**Gross energy (MJ/kg)**	19.8
**Fibre (%)**	0.7
**Ash (%)**	9
**Starch (%)**	15.5
**Phosphorous (%)**	1.5
**Phospholipids (%)**	1.5
**Cholesterol (%)**	0.25

Vit. A (UI/kg) 10 000 10 000 Vit. D3 (UI/kg) 2 000 2 000 Vit. E (mg/kg) 400 350 Vit. C (mg/kg) 1 000 750.

Table S2 Multifactorial parametric ANOVA results for chlorophyll concentration considering treatment and sampling time. Chlorophyll values represent the mean concentration throughout the experimental period for each treatment.

Factor	Reason-F	P-Value	Ch (mg/l)	
**T: Treatment**	42.07	$2x10^{-7}$	**M**	0.214 ± 0.084^{b}
**d: Day**	5.02	4×10^{-4}	**MP**	0.210 ± 0.075^{b}
Interactions			**P**	0.036 ± 0.024^{a}
**Txd**	1.20	2.940	**C**	0.028 ± 0.024^{a}

Values represented as mean ± standard error of the mean (n=3). Different letters indicate significant differences between treatments considering the overall experimental period ($p < 0.05$). The mean comparison test used was Student-Newman-Keuls.

Table S3: Water quality of treatments

	INITIAL			
	**M**	**MP**	**P**	**C**
**NO_3^-**	232.22 ± 12.88^{b}	238.22 ± 8.51^{b}	58.66 ± 3.47^{a}	60.30 ± 2.57^{a}
**PO_4**	25.67 ± 3.32^{b}	26.13 ± 0.32^{b}	3.97 ± 0.57^{a}	3.32 ± 0.35^{a}
**TSS**	398.50 ± 36.01	403.33 ± 28.66	431.67 ± 43.68	415.83 ± 47.73
	**MEAN EXPERIMENT**			
**TAN**	0.06 ± 0.04	0.05 ± 0.04	0.06 ± 0.05	0.05± 0.04
**NO_2^-**	0.03 ± 0.02	0.02 ± 0.01	0.02 ± 0.01	0.02± 0.01
**NO_3^-**	286.49 ± 1.56^{b}	276.04 ± 58.17^{b}	181.32 ± 13.12^{a}	180.09 ± 11.24^{a}
**PO_4**	20.84 ± 2.63^{c}	21.19 ± 0.14^{b}	12.89 ± 2.08^{a}	13.99 ± 0.70^{a}
**TSS**	480.08 ± 55.33	488 ± 60.63	433 ± 82.22	455 ± 81.10

Values are expressed as mg L^{-1} for total ammonia nitrogen (TAN), nitrite (NO_2^-), nitrate (NO_3^-), and phosphate (PO_4^{3-}). Total Suspended Solid (TSS). Values represented as mean ± standard error of the mean (n=3). Different letters indicate significant differences between treatments. ($p < 0.05$).

Table S4: Summary of the quality of 16 rRNA gene sequencing of biofloc and shrimp intestine samples at the beginning and end of the experiment.

		Inputs	Filtered	Inputs passed filter (%)	Denoised	Non-chimeric	Inputs non-chimeric (%)
Biofloc	**Initial**						
	M	67917 ± 20246	64370 ± 19173	94.8 ± 0.1	62091 ± 18978	62091 ± 18978	91.2 ± 0.9
	MP	54914 ± 8138	52138 ± 7782	94.9 ± 0.1	50189 ± 8035	50189 ± 8035	91.2 ± 1.1
	P	71311 ± 9681	66008 ± 9320	92.5 ± 2.1	63292 ± 9275	63292 ± 9275	88.7 ± 2.5
	C	64178± 4941	59033 ± 3457	92.1 ± 3.3	56216 ± 3321	56216 ± 3321	87.7 ± 3.2
	Total	64580	60387	93.6	57947	57947	89.7
	**Final**						
	M	49546 ± 3271	45218 ± 4743	91.3 ± 7.0	43087 ± 5062	43087 ± 5062	86.9 ± 7.1
	MP	65442 ± 12743	62238 ± 12360	95.0 ± 0.5	59929 ± 12113	59929 ± 12113	91.5 ± 0.8
	P	52279 ± 2933	49506 ± 2391	94.7 ± 0.8	47421 ± 2305	47340 ± 2176	90.6 ± 1.0
	C	57022 ± 1511	53480 ± 2847	93.7 ± 2.5	51462 ± 2925	51462 ± 2925	90.2 ± 2.8
	Total	56072	52610	93.7	50475	50455	89.8
	**TOTAL**	60326	56499	93.6	54211	54201	89.8
Shrimp intestine	**Initial**	41155 ± 17164	39124 ± 16365	95.0 ± 0.3	38007 ± 16233	38007 ± 16233	91.1 ± 1.3
	**Final**						
	M	17521 ± 13516	15996 ± 12372	90.5 ± 4.4	14894 ± 12104	14894 ± 12104	81.8 ± 7.6
	MP	15850 ± 14539	14948 ± 13931	93.2 ± 2.3	13781 ± 13414	13780 ± 13413	81.5 ± 8.9
	P	20935 ± 17593	19616 ± 16729	92.4 ± 5.0	18804 ± 16426	17784 ± 15279	81.9 ± 6.4
	C	15016 ± 9646	13679 ± 9005	90.3 ± 4.6	12692 ± 8794	12673 ± 8785	81.2 ± 8.0
	Total	17330	16060	91.6	15043	14783	81.6
	**TOTAL**	22095	20673	92.3	19635	19428	83.6

Table S5. Alpha diversity indexes of phylum and family taxonomic level from biofloc microbiota.

	Treatments	Shannon	Simpson	CHAO1
PHYLUM	**Initial**	1.72 ± 0.09	0.75 ± 0.03	22.25 ± 3.31
	**Final**			
	M	1.81 ± 0.18	0.77 ± 0.05	25.00 ± 1.73
	MP	1.80 ± 0.04	0.76 ± 0.02	23.67 ± 2.31
	P	1.73 ± 0.28	0.75 ± 0.08	21.33 ± 1.53
	C	1.90 ± 0.06*	0.80 ± 0.02*	23.00 ± 1.00
	**Final**			
	Chs	1.80 ± 0.07	0.76 ± 0.03	24.33 ± 0.71
	N-Chs	1.82 ± 0.08	0.78 ± 0.02	22.17 ± 0.69
	Pbs	1.76 ± 0.07	0.75 ± 0.01	22.50 ± 0.78
	N-Pbs	1.86 ± 0.07	0.78 ± 0.02	24.00 ± 0.70
FAMILY	**Initial**	1.66 ± 0.10	0.72 ± 0.04	18.33 ± 0.52
	**Final**			
	M	1.49 ± 0.20	0.64 ± 0.09	15.67 ± 3.01
	MP	1.55 ± 0.22	0.66 ± 0.09	17.88± 3.00
	P	1.71 ± 0.19	0.75 ± 0.07	15.00± 3.92
	C	1.66 ± 0.10	0.72 ± 0.04	14.86 ± 2.48*
	**Final**			
	Chs	1.52 ± 0.20	0.65 ± 0.09	16.93± 3.10
	N-Chs	1.59 ± 0.27	0.71 ± 0.11	14.91 ± 2.88
	Pbs	1.49 ± 0.24	0.65 ± 0.10	16.9 ± 13.45
	N-Pbs	1.61 ± 0.22	0.70± 0.09	15.23 ± 2.65

Values are presented as mean ± standard error of the mean (n=3). Superscript letters in the final values indicate significant differences among them. An asterisk (*) indicates significant differences compared to the initial treatment within the same column ($p < 0.05$). The means comparison test used was the Student–Newman–Keuls test.

Table S6. Alpha diversity indexes of family taxonomic level from shrimp intestine microbiota.

Treatments	Shannon	Simpson	Chao1
**Initial**	2.96 ± 0.05	0.91 ± 0.01	79.17 ± 7.68
**Final**			
M	3.03 ± 0.16	0.90 ± 0.01	61.50 ± 17.09*
MP	3.01 ± 0.41	0.90 ± 0.09	59.25 ± 16.26*
P	2.65 ± 0.49	0.84 ± 0.09	54.25± 21.26*
C	3.01 ± 0.29	0.91 ± 0.01	57.29 ± 16.02*
**Final**			
Chs	3.02 ± 0.32	0.90 ± 0.07	60.21± 16.00*
N-Chs	2.88 ± 0.39	0.88 ± 0.07	56.18 ± 17.20*
Pbs	2.89 ± 0.45	0.88 ± 0.09	57.58 ± 17.35*
N-Pbs	3.02 ± 0.23	0.91± 0.03	59.23 ± 15.96*

Values are presented as mean ± standard error of the mean (n=3). Superscript letters in the final values indicate significant differences among them. An asterisk (*) indicates significant differences compared to the initial treatment within the same column ($p < 0.05$). The means comparison test used was the Student–Newman–Keuls test.

Table S7. PERMANOVA results based on Bray–Curtis dissimilarities showing the effects of treatment on bacterial beta diversity in biofloc and shrimp intestine samples at the phylum and family levels.

			Df	SumOfSqs	R2	F	Pr(>F)
BIOFLOC	Phylum	Initial and Final	4	0.1487972	0.3261045	1.209.774	**0.320**
			10	0.3074896	0.6738955		
			14	0.4562868	10.000.000		
		Final	3	0.09026939	0.2322058	0.8064863	**0.557**
			8	0.29847794	0.7677942		
			11	0.38874733	10000000		
	Family	Initial and Final	4	0.3634827	0.3672214	1450829	**0.198**
			10	0.6263362	0.6327786		
			14	0.9898189	10000000		
		Final	3	0.2172161	0.2622544	0.9479489	**0.479**
			8	0.6110487	0.7377456		
			11	0.8282648	10000000		
INTESTINE	Phylum	Initial and Final	4	0.1614448	0.6020756	3.7826	**0.005**
			10	0.1067022	0.3979244		
			14	0.2681470	1.0000000		
		Final	3	0.08189264	0.4467265	2.153132	**0.108**
			8	0.10142453	0.5532735		
			11	0.18331717	1.0000000		
	Family	Initial and Final	4	0.4515990	0.5220954	2.731169	**0.013**
			10	0.4133751	0.4779046		
			14	0.8649741	1.0000000		
		Final	3	0.1949871	0.3017905	1.152626	**0.324**
			8	0.4511138	0.6982095		

	11	0.6461009	1.0000000

Results of permutational multivariate analysis of variance (PERMANOVA) performed on beta diversity data to evaluate differences in bacterial community structure among experimental treatments in biofloc and shrimp intestine samples (n=3). Analyses were conducted at both phylum and family taxonomic levels using datasets including both sampling times ("Initial and Final") and final sampling time only ("Final") (n=3, 2 shrimp per tank). Degrees of freedom (Df), sums of squares (SumOfSqs), coefficient of determination (R^2), pseudo-F statistic (F), and associated p-values (Pr(>F)) are presented. Significant differences were considered at $p < 0.05$ and are shown in bold.
